# Exogenous Thyropin from p41 Invariant Chain Diminishes Cysteine Protease Activity and Affects IL-12 Secretion during Maturation of Human Dendritic Cells

**DOI:** 10.1371/journal.pone.0150815

**Published:** 2016-03-09

**Authors:** Tina Zavašnik-Bergant, Martina Bergant Marušič

**Affiliations:** 1 Department of Biochemistry, Molecular and Structural Biology, Jožef Stefan Institute, Ljubljana, Slovenia; 2 Tissue Typing Centre, Blood Transfusion Centre of Slovenia, Ljubljana, Slovenia; Istituto Superiore di Sanità, ITALY

## Abstract

Dendritic cells (DC) play a pivotal role as antigen presenting cells (APC) and their maturation is crucial for effectively eliciting an antigen-specific immune response. The p41 splice variant of MHC class II-associated chaperone, called invariant chain p41 Ii, contains an amino acid sequence, the p41 fragment, which is a thyropin-type inhibitor of proteolytic enzymes. The effects of exogenous p41 fragment and related thyropin inhibitors acting on human immune cells have not been reported yet. In this study we demonstrate that exogenous p41 fragment can enter the endocytic pathway of targeted human immature DC. Internalized p41 fragment has contributed to the total amount of the immunogold labelled p41 Ii-specific epitope, as quantified by transmission electron microscopy, in particular in late endocytic compartments with multivesicular morphology where antigen processing and binding to MHC II take place. In cell lysates of treated immature DC, diminished enzymatic activity of cysteine proteases has been confirmed. Internalized exogenous p41 fragment did not affect the perinuclear clustering of acidic cathepsin S-positive vesicles typical of mature DC. p41 fragment is shown to interfere with the nuclear translocation of NF-κB p65 subunit in LPS-stimulated DC. p41 fragment is also shown to reduce the secretion of interleukin-12 (IL-12/p70) during the subsequent maturation of treated DC. The inhibition of proteolytic activity of lysosomal cysteine proteases in immature DC and the diminished capability of DC to produce IL-12 upon their subsequent maturation support the immunomodulatory potential of the examined thyropin from p41 Ii.

## Introduction

Dendritic cells (DC) function as professional antigen-presenting cells (APC) that link the innate and adaptive immune responses against invading pathogens. DC play a key role in antigen-specific immunity [1] and their proteolytic potential is crucial for effective degradation of antigens [2] later to be exposed on the surface of these APC for recognition by specific T-cell receptors [3]. Various cystatin-type inhibitors of cysteine proteases have been suggested to regulate the proteolytic activity of DC and their immunomodulatory properties [4–7]. In this study the effect of a protease inhibitor of another type is illustrated.

Since the discovery of the chaperone invariant chain (Ii) [8], various associated structural and functional features have been reported, with physiological roles beyond its association with major histocompatibility complex class II molecules (MHC II) [9]. Four isoforms (S2 Fig), termed according to their size as p33, p35, p41 and p43, are known in human [10–12]. The shorter (p33 and p35) and longer (p41 and p43) isoforms are distinguished by an additional amino acid sequence present in the latter. Differential regulation of the expression of MHC II, p33 Ii and p41 Ii at the transcriptional level during human DC maturation has been reported [13]. Newly synthesized Ii and MHC II associate into a nonameric complex [14] in which the luminal domains of Ii interact with MHC II [15,16] and prevent premature binding of other peptides. The generated MHC II-Ii complexes are transported through the Golgi and onward into the endocytic pathway of APC [17,18]. MHC II-Ii complexes proceed from the Golgi, first to endosomes or to the plasma membrane, and then back to MHC II-loading compartments. The trimerization domain is cleaved and Ii is degraded sequentially (reviewed in [19–22]). In the moderately acidic milieu (in antigen-processing and MHC II-loading compartments) Ii is replaced by the peptides generated from internalized and proteolytically processed antigens [23–26]. Peptide loading is assisted by human-leukocyte antigen-DM (HLA-DM), which is modulated by another chaperone HLA-DO [27,28]. Besides the role of Ii in Ag processing and presentation [29,30], several other, MHC II-independent, functions have been proposed: involvement of Ii in pro-inflammatory cytokine macrophage migration inhibitory factor (MIF), signaling in association with CD44 [31,32], regulation of DC migration through the binding of Ii and the actin-based motor protein myosin II [33], and a role in activating the NF-κB pathway by Ii intramembrane proteolysis [34]. Another non-redundant role of p41 Ii has been described in lungs of allergen-treated mice, where selective p41 Ii promotion of IgE production proceeds in parallel with the development of airway hyper responsiveness [35].

The inhibitory activity of an additional luminal 64 amino-acid region of p41 Ii is known [36–38]. This p41 fragment, having a characteristic thyroglobulin type-1 domain structure [39], has been classified to a group of protease inhibitors named thyropins [40,41]. However, p41 fragment has long been considered as a specific inhibitor [39] and in APC also as a chaperone [42] of only one particular cysteine protease, i.e., cathepsin L. Endogenous p41 fragment from p41 Ii was shown to stabilize cathepsin L in MHC II-loading compartments in mouse bone-marrow derived APC [42]. Furthermore, p41 fragment is secreted from APC to their extracellular milieu where it preserves the local concentration of functional cathepsin L involved in the degradation of extracellular matrix and the migration of APC during inflammation [43,44].

More recent kinetic studies, performed with an expanded list of isolated recombinant cysteine proteases, have indicated that the p41 isoform of Ii, with its inhibitory thyropin segment, might play a wider role than previously thought [38], i.e., by affecting, in addition, other (endogenous) lysosomal cysteine cathepsins inside the APC, the site of p41 Ii original expression. The latter has not been further investigated in cells and not in human DC in particular.

Further, it is not known what happens to the secreted p41 fragment when it dissociates from the complex with cathepsin L, after the complex is released into the extracellular milieu of APC as reported by [43]. It is not known what processes or which cells could be targeted by this free and still inhibitory active thyropin when reaching and persisting in the extracellular milieu. Interestingly, secretory proteins structurally similar to p41 fragment were recently identified in insect excretions such as tick saliva [45,46]. The saliva is injected into the host skin, where various types of DC reside, facilitating parasite feeding and down-modulates the host immune response [47].

Possible effects of exogenous p41 fragment or related thyropin inhibitors acting on human immune cells have not been reported. Here we have investigated whether exogenous p41 fragment possesses the ability to change the characteristics of targeted human DC associated with their efficient antigen presentation function. We report that cysteine protease activity within the endocytic pathway of human immature DC is diminished on successful internalization of an exogenous thyropin inhibitor and that p41 fragment reduces the secretion of interleukin-12 during the subsequent maturation of treated DC.

## Materials and Methods

### Ethics statement

Investigations concerning human cells/lymph node tissue, reported in the study entitled “Exogenous thyropin from p41 invariant chain diminishes cysteine protease activity and affects IL-12 secretion during maturation of human dendritic cells”, were approved by the National Medical Ethics Committee of the Republic of Slovenia (Document No. 51k/02/02). Lymph-node tissue (un-invaded by tumor cells) was obtained from two patients undergoing mastectomy and auxiliary dissection for breast carcinoma at the Institute of Oncology, Ljubljana, Slovenia. The entire study was retrospective. All samples (sections of tissue) had been obtained previously for diagnostic purposes. No additional intervention or investigation of patients was either required or performed in the reported research. The anonymity of the patients was assured. The monocytes used in the reported study were derived from buffy coats obtained from healthy blood donors (volunteers), and provided anonymously by the Blood Transfusion Centre of Slovenia, Ljubljana, Slovenia. According to Slovene legal requirements, i.e., “Supply of Blood Act” (ZPKrv-1, UL RS No.104/06) and “Rules concerning the method of storing, delivery, transport and elimination/removal of unused blood and blood preparations” (UL RS No. 100/02, Article 17 and UL RS No. 104/06 –ZPKrv-1), stored unused blood and blood products and blood samples that were left after the testing can, in agreement with the doctor responsible, be devoted to laboratory purposes or for research that has been approved by the Medical Ethics Committee (UL RS No. 100/02, Article 17). For this reason, written informed consent for the use of buffy coats (as a by-product of blood processing of donated blood) for the research reported here, was not additionally obtained from the healthy blood donors (volunteers) by the Blood Transfusion Centre of Slovenia, Ljubljana. The procedure applied in the use of the buffy coats for the research reported here was approved by the National Medical Ethics Committee of the Republic of Slovenia (Document No. KME 67/12/00, linked to Document No. KME 77/01/00 and Document No. KME 86/02/00).

### Antibodies

Monoclonal antibody (mAb) against human p41 Ii (clone 1D12) and polyclonal antibodies (pAb) against cathepsins S and L (S5 Fig) and H were applied as described [48,49]. Mouse mAbs against LAMP-2 (H4B4 mAb), HLA-DR (TÜ36 mAb), HLA-DM (MaP.DM1 mAb) and Ii (anti-CD74, LN2 mAb) were from BD Biosciences, and mouse anti-CD68 mAb (KP1) from DAKO Agilent Technologies. Highly cross-adsorbed goat anti-mouse IgG and goat anti-rabbit IgG (both labelled with Alexa Fluor 488 or Alexa Fluor 546), from Life Technologies-Molecular Probes, were used as secondary antibodies. Rabbit-anti mouse IgG antibody, from MP Biomedicals, was used as bridging antibody. Rabbit polyclonal anti-NF-κB p65 antibody, ab7970, was from Abcam. All commercial antibodies were used according to the suppliers’ recommendations.

### Monocyte-derived immature and mature DC

Immature and mature DC were generated from human monocytes. Human monocytes were isolated from buffy coats of healthy donors (Blood Transfusion Centre of Slovenia, Ljubljana) and further differentiated to immature DC *in vitro* at 5×10^5^ cells/ml, using GM-CSF (Leucomax, 500 U/ml, Novartis Pharma) and IL-4 (400 U/ml) for five days as described [48,50]. Immature DC (1×10^6^ cells/ml) were matured, either with TNF-α (15 ng/ml, R&D Systems) and GM-CSF (Leucomax, 1000 U/ml, Novartis Pharma) for three to five days or with LPS (20 ng/ml, Sigma-Aldrich) and GM-CSF (Leucomax, 500 U/ml, Novartis Pharma) for up to 48 h. Cell viability was checked using trypan blue (Sigma-Aldrich). Alexa Fluor 546-labelled dextran (MW 10.000, Life Technologies-Molecular Probes) was added, at 10 μg/ml and 100 μg/ml, to DC and incubated for 40 min at 37°C. In a control experiment, cells were preincubated for 30 min at 4°C to slow the metabolic uptake of the dextran conjugate.

### Lymph node tissue

Paraffin sections (5 μm) of lymph node tissue were labelled with anti-p41 Ii mAb as described [49]. Total tissue lysate was prepared from non-fixed tissue previously frozen in liquid nitrogen. Pieces of the latter, in 0.01 M phosphate buffer (pH 7.2), were sonicated using a Branson Digital Sonifier W-450 (Branson). The non-soluble fraction was pelleted by centrifugation. The supernatant containing soluble proteins was dialyzed in 0.01 M phosphate buffer using Microcon YM-3 (Millipore). Proteins were separated on SDS-PAGE and checked for the presence of p41 Ii.

### Differentiated MUTZ-3 cells and NF-κB labelling

Human CD34+ acute myeloid leukemia cell line MUTZ-3 (catalogue no. ACC-295) was from Leibniz Institute DSMZ-German Collection of Microorganisms and Cell Cultures (Germany). Cells were grown in α-MEM with 20% heat-inactivated FBS (PAA Laboratories–GE Healthcare Life Sciences), 1% Glutamax (Life Technologies) and 40 ng/ml (320 IU/ml) GM-CSF (CellGro) as described [51]. MUTZ-3 cells were differentiated to immature DC at 5×10^5^ cells/ml, using 62.5 ng/ml (500 IU/ml) GM-CSF, 100 ng/ml (500 IU/ml) IL-4 and 2.5 ng/ml (25 IU/ml) TNF-alpha (all from CellGro) for 4 days. Differentiated MUTZ-3 cells were pretreated with 3.5 μM p41 fragment for 4 h and then stimulated with 20 ng/ml LPS (Sigma-Aldrich) for 2 h. Further, following the preincubation with 10 μM NF-κB SN50 (cell-permeable inhibitor peptide of NF-κB nuclear translocation) or 10 μM NF-κB SN50M (cell-permeable inactive control peptide for SN50), stimulated cells (20 ng/ml LPS) were fixed, immunolabelled with anti-NF-κB p65 antibody and analyzed by confocal microscopy. SN50 and SN50M were from Calbiochem (Merck Millipore).

### SDS-PAGE, isoelectric focusing (IEF), native PAGE and Western blotting

Isolated p41 fragment, isolated p41 fragment in complex with cathepsin L, recombinant p41 Ii without cytoplasmic and transmembrane domain (with inhibitory p41 fragment included), recombinant p31 Ii without cytoplasmic and transmembrane domain (and no inhibitory fragment) [49], recombinant cathepsins L and S [38], cell lysates and lymph node lysate were applied to SDS-PAGE or to IEF (pH 3–9). p41 fragment and p41 fragment in complex with cathepsin L were also subjected to native PAGE at pH 5.0. Prior to the latter, samples were incubated overnight with 1 U of N-glycosidase F (Sigma-Aldrich) per 10 μg of protein. For Western blotting proteins were transferred onto PVDF membrane (Millipore) and labelled with primary anti-p41 Ii mAb, anti-cathepsin S pAb or anti-cathepsin L pAb and HRP-conjugated secondary antibody (Jackson ImmunoResearch). In addition, anti-Ii mAb (clone LN2) was used to label both recombinant Ii isoforms (S4 Fig).

### Flow cytometry

Differentiation and maturation of cells were followed and evaluated by surface expression of CD1a, CD14, CD40, CD54, CD80, CD83, CD86 and HLA-DR using fluorescently conjugated primary antibodies (BD Biosciences). Total p41 Ii content of DC was followed during their maturation. DC were fixed with 4% paraformaldehyde (Sigma-Aldrich) and labelled with anti-p41 Ii mAb, followed by Alexa Fluor 488-labelled secondary antibody. Isotype matched mouse immunoglobulins (Sigma-Aldrich) were used instead of anti-p41 Ii mAb (negative control). 10.000 cells were gated per sample and evaluated for specific labelling using BD FACSCalibur^™^ flow cytometer (BD Biosciences) and BD CellQuest^™^ software.

### Confocal microscopy

DC were cytocentrifuged onto glass slides coated with poly-L-lysine (Sigma-Aldrich). They were fixed with 4% paraformaldehyde and permeabilized with 0.1% Triton X-100. Non-specific binding was blocked with BSA. DC were labelled with anti-p41 Ii mAb, followed by Alexa Fluor 488-labelled goat anti-mouse IgG antibody. In cases of double labelling, species-specific Alexa Fluor 546-conjugated secondary antibody was used. Controls were run in the absence of either primary and secondary antibody. Labelled optical sections were examined using confocal laser scanning microscopes Carl Zeiss LSM 510 and Leica TCS SP5 X. Fluorophores Alexa Fluor 488 and Alexa Fluor 546 were excited with 488 nm and 543 laser lines, respectively. Carl Zeiss LSM image software was used to evaluate the co-localization of p41 Ii and other labelled intracellular proteins (cathepsins H, L and S, HLA-DR, HLA-DM, CD68, LAMP-2).

### Internalization of p41 fragment

Inhibitory p41 fragment and cysteine protease cathepsin L were isolated from human kidney as a complex which was then dissociated as reported [36,37]. When used in cell studies, isolated p41 fragment in PBS was filtered through a 0.22 μm Durapore PVDF membrane/Millex GV filter unit (Millipore) and additionally purified using Detoxi-Gel^™^ Endotoxin Removing Resin (Thermo Scientific). Prior to study of its internalization the inhibitory activity of p41 fragment was determined using active site titration of recombinant human cathepsin L. Active cathepsin L (2.5 nM) was incubated with various concentrations of p41 fragment (0.5 nM– 3 nM). AMC release from Z-Phe-Arg-AMC substrate (Bachem) was measured [38, 52, 53] and related to p41 fragment/cathepsin L molar ratio.

Optionally, and prior to its internalization into immature DC, exogenous p41 fragment (14 kDa) was fluorescently labelled with Alexa Fluor 488 dye (Alexa Fluor 488 Microscale Protein Labelling Kit, Life Technologies-Molecular Probes). Unbound Alexa Fluor 488 dye (643 Da) was removed from the reaction mixture by gel filtration (size-exclusion chromatography) exactly as recommended by the producer. Additional purification was performed by two successive dialyses (and filtrations) using Microcon Centrifugal Filter Device (Millipore). Before each filtration, 100 μl of PBS was added to 100 μl of conjugated p41 fragment in PBS in the upper sample reservoir. Diluted sample (200 μl) was filtered using Microcon YM-3 (MWCO 3 kDa). Small aliquots (equal volumes) were taken from 100 μl of the retentate in the upper sample reservoir (containing conjugated protein and remaining unbound dye in PBS) and from 100 μl of the filtrate in the lower sample reservoir (containing unbound dye in PBS). Fluorescence of samples, taken after each purification step, was measured with Tecan microplate reader (ex. 495 nm/em. 519 nm). Immature DC (1×10^6^ cells/ml) were cultured in the presence of exogenous human p41 fragment (0.035 μM, 0.35 μM and 3.5 μM). After 6 h the culture medium was exchanged with fresh medium, i.e., RPMI-1640 medium (Lonza) with 10% FCS (PAA Laboratories), without inhibitory p41 fragment. DC were then matured with TNF-α for 3 days or with LPS for 48 h. As for the non-treated DC no p41 fragment was added prior to their maturation. At selected time points cell-free supernatants (for the determination of secreted IL-12) and cells (for the determination of residual cysteine protease activity) were collected. In addition, pelleted immature DC, grown in the presence of 3.5 μM p41 fragment for 6 h, were fixed and prepared as an additional sample for immunogold labelling and transmission electron microscopy (TEM).

### Ultrathin cryosections, immunogold labelling and transmission electron microscopy

DC were fixed and ultrathin cryosections prepared exactly as described [54]. Sections were incubated with anti-p41 Ii mAb, followed by (bridging) rabbit-anti mouse antibody (MP Biomedicals). Cathepsin S distribution was evaluated using rabbit anti-cathepsin S polyclonal antibody. Sections were incubated with Protein A–10 gold according to the producer’s instructions (Cell Microscopy Centre, University Medical Centre Utrecht). Primary antibody was omitted in controls. Finally, proteins were contrasted with 0.4% uranyl acetate (SPI-ChemTM uranyl acetate, SPI Supplies) in solution with 1.8% methyl cellulose (Sigma-Aldrich). TEM was performed with a Philips CM120 BioTWIN transmission electron microscope at 100 kV. Micrographs were taken using a KeenView digital camera and iTEM software.

### Quantification of immunogold distribution with chi-squared (X^2^) test

Immunogold distributions were evaluated as described [55]. Random sampling of a specimen was followed by counting approximately 200 gold particles to indicate the positions of labelled p41 Ii and cathepsin S in various cell compartments. Immunogold distribution in DC preincubated with p41 fragment (disclosing the localization of successfully internalized exogenous p41 fragment and endogenously expressed p41 Ii) was compared to that in non-treated DC (with labelled endogenous p41 Ii only). Chi-squared (X^2^) test was used to test the null hypothesis of no difference in subcellular localization of particular labelled protein (p41 Ii or cathepsin S) in the same set of compartments in different cell populations (see S1 Table). The predicted number of gold particles on a given compartment in a given cell group was calculated by contingency table analysis as [column total × row total)/grand total]. The grand total was the sum of the column (or row) totals. For each compartment and cell type, the partial chi-squared value (X^2^) was given by [(observed−expected gold particles)^2^/expected gold particles].

### Ultrastructure of DC populations

DC were prepared for ultrastructure observation by TEM according to described protocols [56]. DC were fixed with 1% glutaraldehyde, post-fixed and stained with 1% osmium tetraoxide to contrast membranes and post-fixed and stained with 1% uranyl acetate for contrasting of proteins. Cells were dehydrated stepwise in ethanol series and infiltrated and embedded in epoxy resin. Polymerized blocks with pelleted cells were trimmed and cut into ultrathin sections. Samples on grids were observed at 100 kV with a Philips CM120 BioTWIN transmission electron microscope. Three DC populations were compared: (1) immature DC, (2) mature DC after 3 days of maturation with TNF-α and (3) mature DC, preincubated with p41 fragment (3.5 μM) for 6 h prior to onset of maturation, and then matured with TNF-a for 3 days.

### Protease activity of DC lysates

Immature DC (1×10^6^ cells/ml), grown for 6 h in the presence of p41 fragment (0.035 μM, 0.35 μM and 3.5 μM), were pelleted and rinsed vigorously in 0.1 M phosphate buffer (pH 6.0). DC were sonicated on ice using a Branson Digital Sonifier W-450 (Branson). Proteases inhibitor cocktail (Sigma-Aldrich) was added (1:100) only when lysates were devoted to SDS-PAGE analysis. Non-soluble fractions were pelleted and removed by centrifugation at 16.000 g at 4°C. Soluble fractions (DC lysates in phosphate buffer) were kept at -80°C. Total protein content was determined by standard Bradford protein assay (Bio-Rad). Optionally, 10 μM E-64 (Sigma-Aldrich), a synthetic inhibitor of cysteine proteases, was added to the cell lysate prior to protease activity measurement. Sterile PBS was added to non-treated cells instead of p41 fragment. The residual cysteine protease activity against 10 μM fluorogenic substrate Z-Phe-Arg-AMC (Bachem) was measured as reported [38,52,53]. Released AMC was evaluated as dF/dt with AMC excitation at 370 nm and emission at 460 nm. An increase in fluorescence signal was measured with a Tecan Safire plate reader (Tecan).

### Quantification of IL-12 (p70) secretion during DC maturation

Immature DC were preincubated with p41 fragment (0.035 μM, 0.35 μM and 3.5 μM) for 6 h prior to their maturation with TNF-α or LPS. Alternatively, immature DC were preincubated with two recombinant Ii isoforms (with or without inhibitory p41 fragment) and matured with TNF-α (S4 Fig). Sterile PBS was added to non-treated cells instead of p41 fragment or recombinant Ii. Cell-free supernatants were collected at up to 3 days of maturation with TNF-α and up to 2 days of maturation with LPS. 500 μl samples, each with 5×10^5^ cells, were collected at 12 h, 23 h, 30 h, 48 h and 72 h. Supernatants were diluted in PBS and concentrations of secreted IL-12 (p70) measured with ELISA (Pierce Endogen). According to the producer’s specifications, this ELISA is specific for the measurement of biologically active human IL-12 (p70); p40 does not cross-react or interfere with the assay. The assay range was from 15 to 600 pg/ml.

## Results

### Characterization of DC

Human monocyte-derived DC (S1 Fig) were used for assessing the effects of exogenous p41 fragment internalized via the endocytic pathway of DC prior to their maturation. Monocyte isolation and DC maturation were repeated as independent biological experiments. The immature DC population was CD1a^+^, CD40^+^, CD54^+^, HLA-DR^+^ and CD14^−^. Surface expression of HLA-DR, CD80, CD83 and CD86 was highly up-regulated during DC maturation and reached a peak after a 3-day incubation with TNF-α. The observed decrease in CD14 surface expression in immature DC, as well as increases of HLA-DR, CD40, CD54, CD80, CD83 and CD86 in mature DC were comparable with those reported [50], confirming the validity of the applied *in vitro* system. Immature DC showed high internalization ability at both dextran concentrations used and fluorescently labelled vesicles were observed throughout the cytoplasm. In mature DC the endocytosis of dextran was reduced but not completely abolished (S1 Fig). Weak fluorescence was still observed in clustered vesicles near the nucleus. No internalized dextran was detected in the control experiment at 4°C. The immature DC included in the following experiments were neither necrotic nor apoptotic (S1 Fig).

### Characterization of anti-p41 Ii mAb

Anti-p41 Ii mAb (clone 2C12 mAb) has been reported to describe the distribution of p41 Ii in lymph node tissue [49]. Here the characterization of another anti-p41 Ii mAb (clone 1D12) is described (S2 Fig). Its selection depended upon its applicability and efficacy for immunogold labelling of p41 Ii in DC prepared as ultrathin cryosections for TEM. It was checked for specific binding to Ii isoform containing p41 fragment and to p41 fragment in complex with the lysosomal protease cathepsin L under denaturing (SDS-PAGE) and non-denaturing (native PAGE) conditions. We also checked whether the presence of glycosylation affects the binding of anti-p41 Ii mAb and whether p41 Ii can be recognized in complex biological samples such as lymph node lysate. The antibody was shown to recognize p41 fragment alone (at 14 kDa), as part of a p41 Ii protein (at 30 kDa), and as a p41 fragment in a complex with cathepsin L (at 32 kDa) (S2 Fig). Anti-p41 Ii mAb also recognized p41 fragment following native PAGE at acidic pH (thereby mimicking the conditions in acidic vesicles in the endocytic pathway of APC). Glycosylation did not affect p41 epitope recognition. In lymph node lysates the mAb detected proteins with molecular masses of 14 kDa, 30 kDa and 43 kDa, corresponding to two p41 fragments and the whole p41 Ii (S2 Fig).

### Endogenous p41 Ii content and its localization during DC maturation

**Fig 1:**
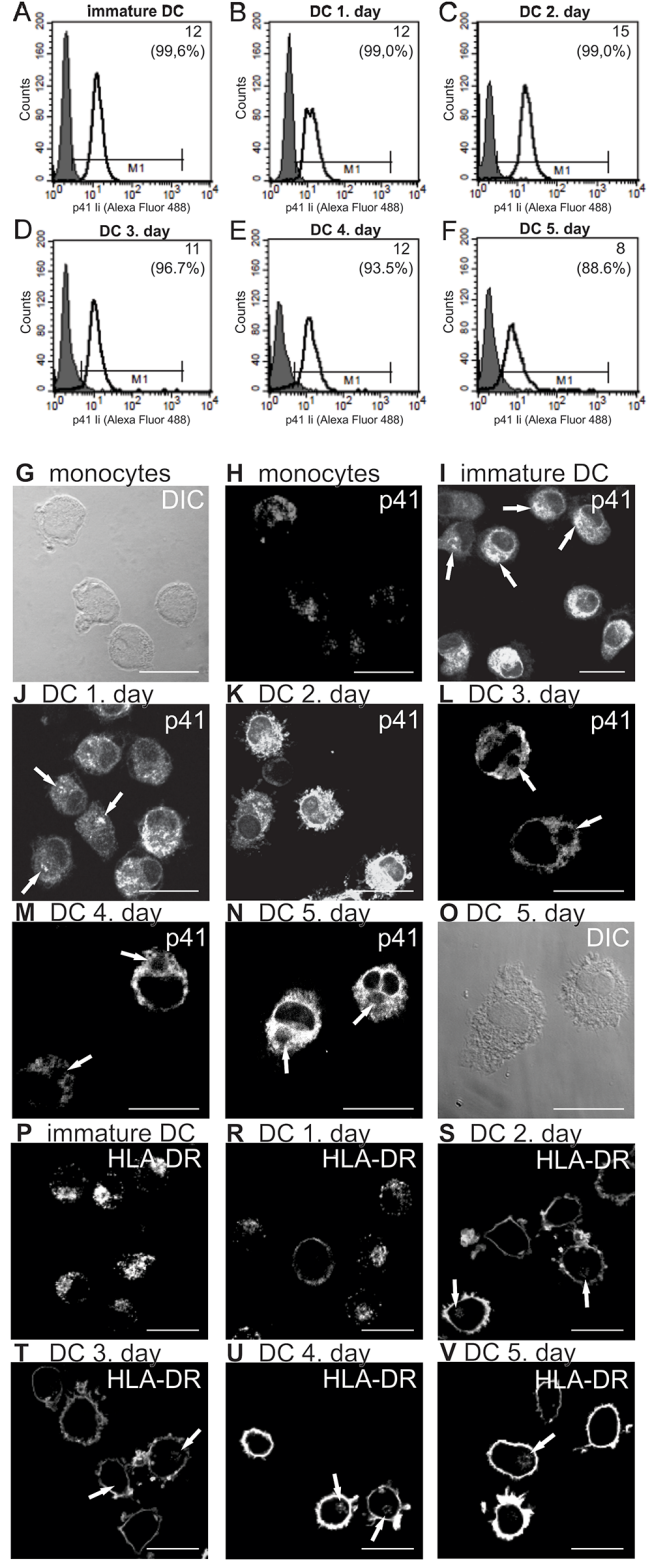
Content (A–F) and localization (G–V) of endogenous p41 Ii and MHC II during maturation of DC with TNF-α. Continuous-line histograms describe the binding of anti-p41 Ii mAb to endogenous p41 Ii. Shadowed histograms represent the corresponding negative controls. Each M1 interval excludes 95% of cells from the corresponding negative control. The percentage of p41 Ii-positive cells in a particular M1 interval and their mean fluorescence intensity (MFI) are stated. A representative analysis of three independent biological repetitions is shown. Confocal images: (H–N) endogenous p41 Ii, (P–V) MHC II (HLA-DR). Bars: 15 μm.

**Fig 2:**
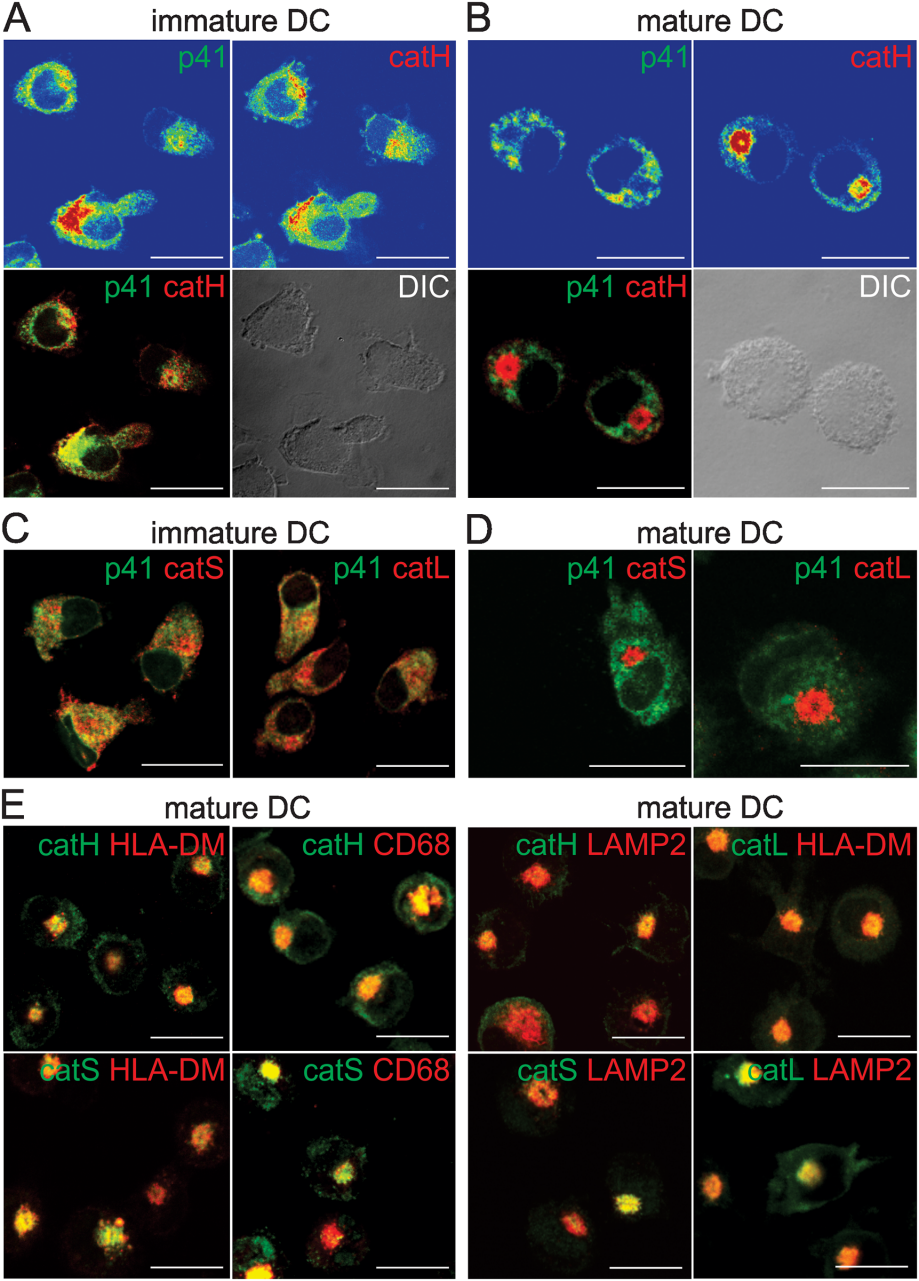
Colocalization of endogenous p41 Ii and lysosomal cysteine cathepsins S, L and H. Confocal images show double immunofluorescence and localization of labelled endogenous p41 Ii, cathepsin H, cathepsin S, cathepsin L, CD68, LAMP-2 and HLA-DM in immature DC (A, C) and in mature DC after a 3-day maturation with TNF-α (B, D, E). Fluorescence intensities (A, B) are presented with a rainbow color palette (blue–the lowest intensity, red–the highest intensity). Only merged images are shown elsewhere (C, D, E).

The epitope recognized by anti-p41 Ii mAb was present in isolated p41 fragment as well as in all endogenous Ii isoforms containing this extra sequence. Therefore, to discriminate the latter from the specific effect of added (exogenous) p41 fragment, the content and distribution of p41 Ii primarily expressed in DC was first determined (Figs 1 and 2).

A small increase in total endogenous p41 Ii content was observed half way through DC maturation (Fig 1C). These results complement those obtained on p41 Ii mRNA at transcription level during DC maturation [13]. Further, the localization of p41 Ii in DC was observed by immunofluorescence confocal microscopy. In monocytes (Fig 1H), labelled vesicles were observed throughout the cytoplasm. In addition to dispersed p41 Ii^+^ vesicles in immature DC, labelling was also evident in endoplasmic reticulum and Golgi (Fig 1I, arrows). In mature DC one discrete location inside the cytoplasm remained unstained with anti-p41 Ii mAb (Fig 1L–1N, arrows). In contrast to p41 Ii, the translocation of MHC II (HLA-DR) associated with DC maturation (S1 Fig) was observed to take place from intracellular vesicles in immature DC (Fig 1P) to the cell surface in mature DC (Fig 1T–1V). A minor portion of MHC II was still retained in clustered p41 Ii^−^ vesicles near the nucleus of mature DC (Fig 1S–1V, arrows). DC grown in the presence of TNF-α beyond 3 days were not included in subsequent double immunolabelling experiments or in the preparation of samples for electron microscopy and ELISA.

### Colocalization of endogenous p41 Ii with endosomal/lysosomal proteases

p41 Ii^+^ vesicles in immature DC were distributed evenly throughout the cytoplasm. No clustering of p41 Ii^+^ vesicles was observed (Fig 2A). Cathepsins were located primarily in dispersed CD68^+^ LAMP-2^+^ HLA-DM^+^ vesicles (in lysosomes and late endosomes), which were found clustered perinuclearly as maturation progressed (Fig 2E). Significant co-localization of endogenous p41 Ii with potential target enzymes (cathepsins S, L and H) was observed in immature DC (Fig 2A and 2C), but not in mature DC (Fig 2B and 2D).

### Active site titration of cathepsin L with p41 fragment

**Fig 3:**
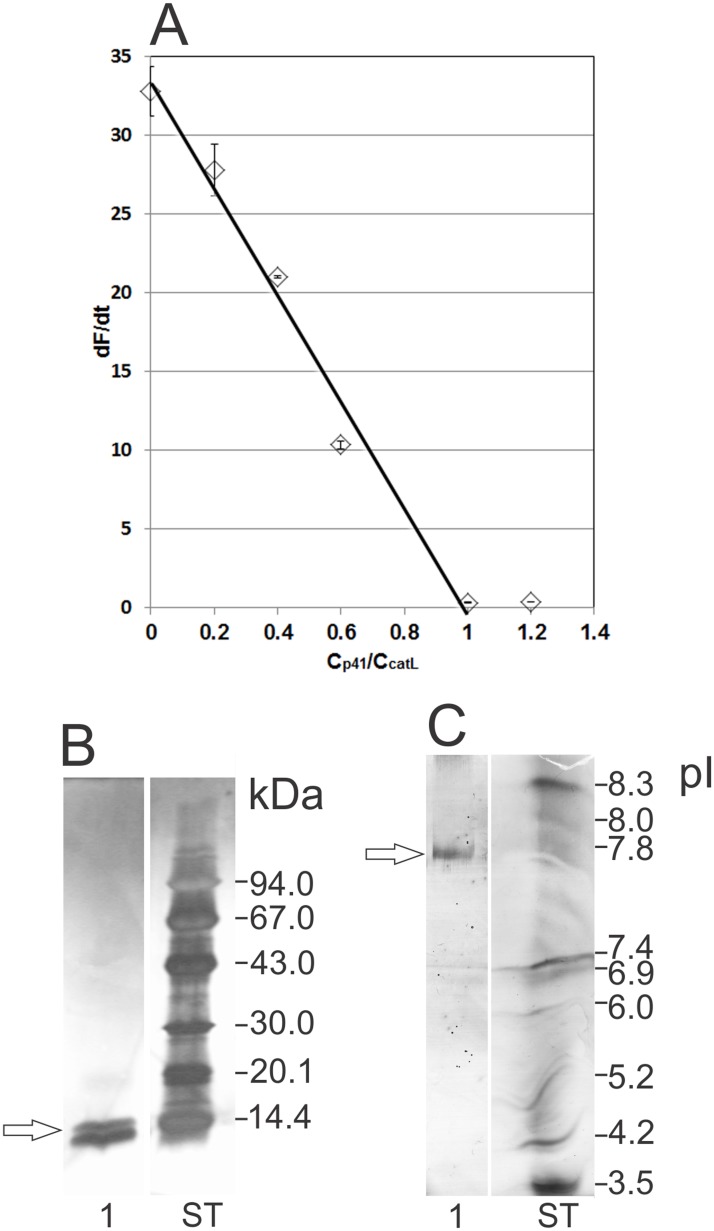
Active site titration of cathepsin L (2.5 nM) with inhibitory p41 fragment (0.5 nM to 3 nM). Released fluorescence was measured in duplicate, average values ± SD are shown. (A) Released fluorescence (dF/dt) is related to p41 fragment/cathepsin L molar ratio. (B) SDS-PAGE and (C) IEF of isolated inhibitory p41 fragment (both stained with silver). ST–standards.

Prior to experiments with DC, the inhibitory activity of isolated p41 fragment was verified by using active site titration of recombinant cathepsin L (Fig 3). Degradation of Z-Phe-Arg-AMC substrate by cathepsin L was abolished at 1:1 molar ratio of p41 fragment and cathepsin L (Fig 3A), thereby verifying that the p41 fragment was inhibitory active when added to immature DC. Isolated inhibitory p41 fragment was detected at 14 kDa and pI 7.8 (Fig 3B and 3C). No other bands, that would indicate the presence of impurities, were observed on silver-stained SDS-PAGE or IEF gel. This p41 fragment was used in subsequent studies with cells.

### Clustering of vesicles in p41 fragment-treated DC was unaltered on their subsequent maturation

**Fig 4:**
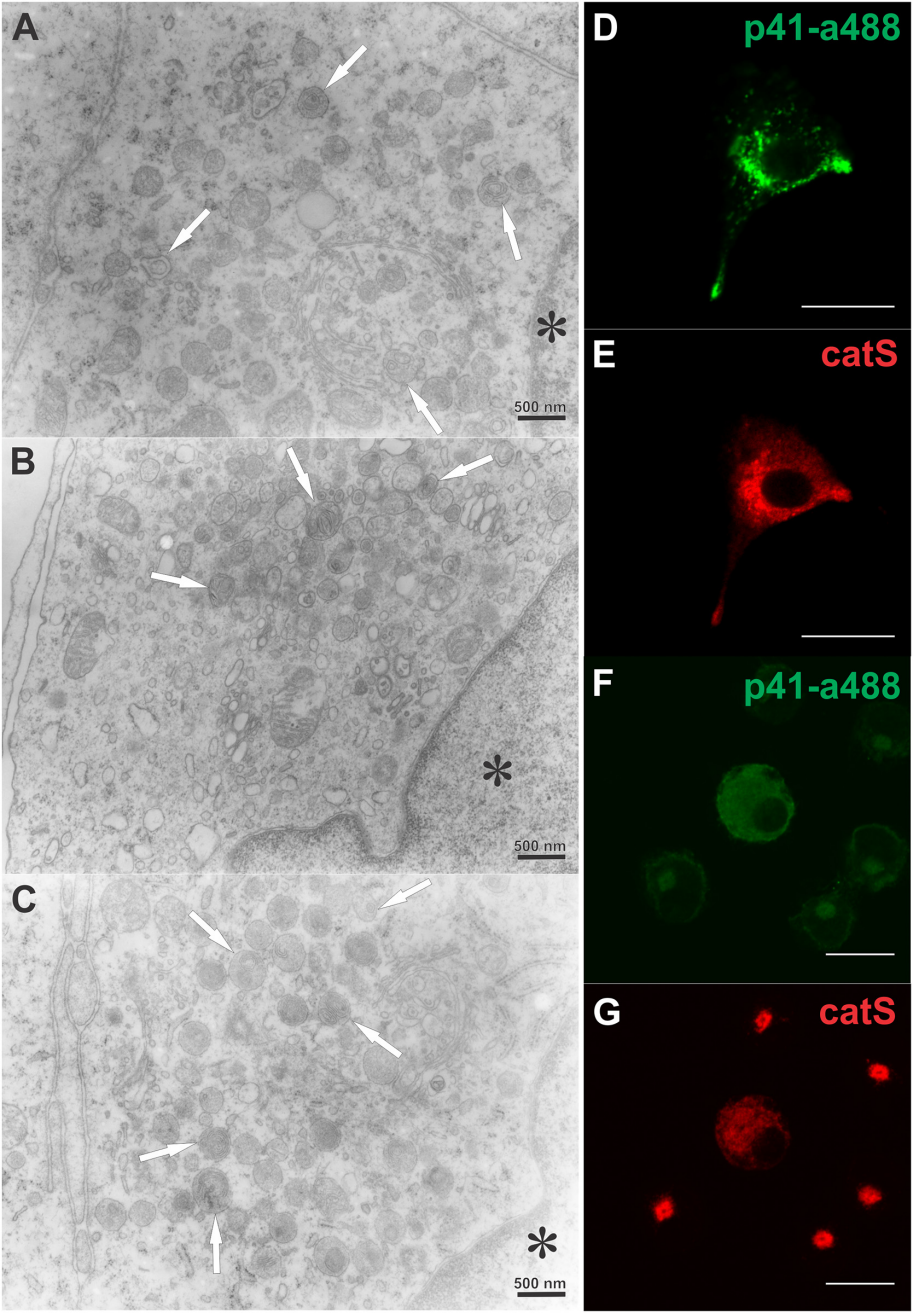
Perinuclear clustering of vesicles in p41 fragment-treated and matured DC. TEM micrographs: (A) immature DC, (B) mature DC after a 3-day maturation with TNF-α and (C) mature DC, preincubated with 3.5 μM p41 fragment for 6 h prior to their maturation. The positions of nuclei are denoted (*). Bars: 500 nm. Confocal images: (D, F) internalized exogenous p41 fragment (conjugated to Alexa Fluor 488) and (E, G) endogenous cathepsin S (labelled with anti-cathepsin S antibody) in treated immature DC after 6 h (D, E) and in subsequently matured DC after 3 days (F, G). Bars: 15 μm.

TEM micrographs of different DC populations were compared to establish whether the introduction of additional (exogenous) p41 fragment affected the intracellular morphology of treated DC (Fig 4). In immature DC (Fig 4A) late endocytic vesicles with abundant multivesicular morphology/inner membranes (multivesicular bodies, MVB) were dispersed throughout the cytoplasm. In contrast, in mature DC these vesicles were clustered together (Fig 4B), most probably concentrating around the microtubule organizing center (MTOC) near the cell nucleus. No difference in morphology of the various vesicles and organelles was observed between DC pretreated with exogenous p41 fragment (Fig 4C) and routinely matured DC (Fig 4B). The clustered vesicles, observed with TEM (Fig 4B and 4C), are taken to correspond to fluorescently immunolabelled vesicles of mature DC that were strongly positive for cathepsins H, L and S, HLA-DM, LAMP 2 and CD68 (Fig 2E) and partly also for MHC II (Fig 1T) but not (endogenous) p41 Ii (Fig 2B and 2D).

### Internalization of fluorescently labelled exogenous p41 fragment

Fluorescently labelled p41 fragment was effectively separated from the unbound Alexa Fluor 488 dye after three steps of purification; gel filtration and two dialyses (S3 Fig). After the second dialysis (and membrane filtration), the fluorescence of unreacted dye in fraction F was minor (530 a.u.) compared to the fluorescence of the fraction E containing the conjugated protein (21,700 a.u.). Further, compared to the preincubation of cells with 3.5 μM p41 fragment (Fig 4D, fluorescently labelled p41 fragment from fraction E), no pronounced fluorescence was observed within the vesicles around the nuclei of treated cells when preincubated (under the same conditions) with the equal volume of fraction F (S3 Fig). The latter contained the remnants of unbound dye but not the conjugated protein after the final purification step (S3 Fig). Thus, it was concluded that conjugated p41 fragment was applicable and was, therefore, used for the colocalization study with cathepsin S.

Internalization of added p41 fragment, previously conjugated to Alexa Fluor 488 dye, was followed, in parallel with TEM, from the culture medium into the endocytic pathway of immature DC. Fluorescently labelled p41 fragment was found in dispersed vesicles (Fig 4D) and co-localised with one of its potentially targeted proteases, cathepsin S (Fig 4E). After a 3-day maturation with TNF-α, cathepsin S-positive vesicles were clustered in the vicinity of the cell nucleus (Fig 4G), independently of DC pretreatment with p41 fragment. The cell with dispersed cathepsin S staining shown in Fig 4G had not been successfully matured after pretreatment with fluorescently labelled p41 fragment.

### Evaluation of p41 Ii and cathepsin S by TEM

**Fig 5:**
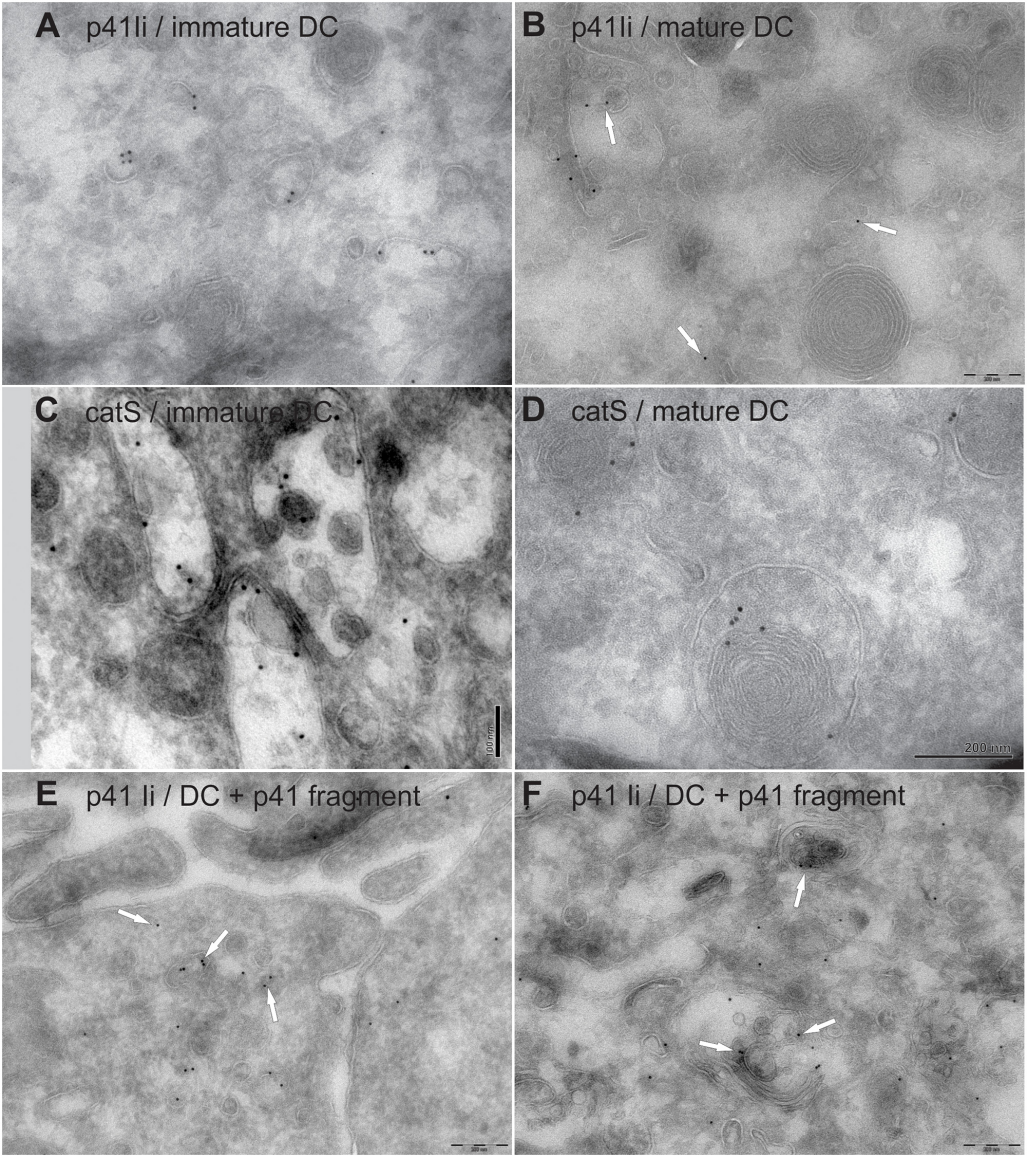
Localization of immunogold labelled p41 Ii (A–B, E–F) and cathepsin S (C–D). TEM micrographs: immature DC (A, C), mature DC after a 3-day maturation with TNF-α (B, D) and immature DC treated with inhibitory p41 fragment (3.5 μM) for 6 h (E, F). Membranes appear white (non-contrasted). Bars: 200 nm (A–B, D, E–F) and 100 nm (C).

The subcellular distribution of endogenous p41 Ii was determined (Fig 5) and compared in different populations of vesicles/organelles in immature and mature DC (Fig 5A and 5B, S1 Table). In the next step, internalization of p41 fragment and its contribution to the total p41 Ii epitope (as well as to endogenous p41 Ii) was determined by specific anti-p41 Ii antibody and gold (Fig 5E and 5F, S1 Table). The distribution of endogenous p41 Ii was compared with that of lysosomal cysteine protease cathepsin S (Fig 5C and 5D, S1 Table).

#### Subcellular distribution of immunogold labelled endogenous p41 Ii

Strong immunogold labelling of p41 Ii in small vesicles and in vesicles with abundant inner membranes (MVB) was observed in non-treated immature and in mature DC (S1 Table). In mature DC 50% of counted gold particles were found in smaller vesicles compared to 41% in the morphologically same type of vesicles in immature DC. In contrast, 20% of gold particles were associated with MVB in immature DC compared to 16% with MVB in mature DC.

The large proportion of gold particles associated with p41 Ii in MVB in immature DC is in line with the co-localization of endogenous p41 Ii and cathepsins S, L and H in immature DC obtained by immunofluorescence microscopy (Fig 2). Conversely, confocal images of mature DC revealed that endogenous p41 Ii-specific fluorescence signal was abolished in strongly LAMP2^+^ CD68^+^ HLA-DM^+^ and weakly HLA-DR^+^ vesicles clustered near the nucleus and also containing the majority of all three labelled cathepsins (Fig 2).

#### Distribution of cathepsin S

The distribution of cathepsin S was clearly different from that of endogenous p41 Ii. Almost 80% of gold particles were associated with MVB in mature DC compared with 71% in immature DC. Further, gold particles were present in small vesicles (18% in immature DC and 11% in mature DC). Weak immunogold labelling of mitochondria, nucleus and cytoplasm was also observed although, at this point, no biologically meaningful explanation can be provided. The intracellular localization of cathepsin S in immature DC did not differ statistically from that in mature DC (total chi-squared value of 6.87, 4 degrees of freedom, P = 0.141) (S1 Table).

#### Contribution of internalized p41 fragment to the total immunogold labelled p41 epitope

Internalization of exogenous p41 fragment into immature DC contributed to the total labelled p41 Ii in these DC. The most evident increase of labelling was observed in peripheral vesicles near the plasma membrane (Fig 5E, arrows) and in MVB (Fig 5F, arrows). This confirms the successful transport of internalized p41 fragment through the endosomal/lysosomal pathway of immature DC, i.e., from early endosomes to more acidic compartments. Immunogold labelled DC populations were compared using the chi-squared (X^2^) test (S1 Table). The subcellular distributions of immunogold labelled p41 Ii in immature and in mature DC did not differ significantly (total chi-squared value of 4.58 and 6 degrees of freedom, P = 0.596). In contrast, when DC were incubated with p41 fragment, the distribution of p41 Ii differed significantly from those of endogenous p41 Ii in immature and mature DC. The gold counts in p41 fragment-treated DC were consistent with a shift towards greater-than-predicted labelling of small vesicles near the plasma membrane (with a partial chi-squared value of 8.33) and of MVB (with a partial chi-squared value of 9.71). The distributions of p41 Ii in immature, mature and treated DC were thus significantly different (P<0.001) for a total chi-squared value of 57.21 and 12 degrees of freedom.

#### Diminished activity of cysteine proteases following internalization of p41 fragment

**Fig 6:**
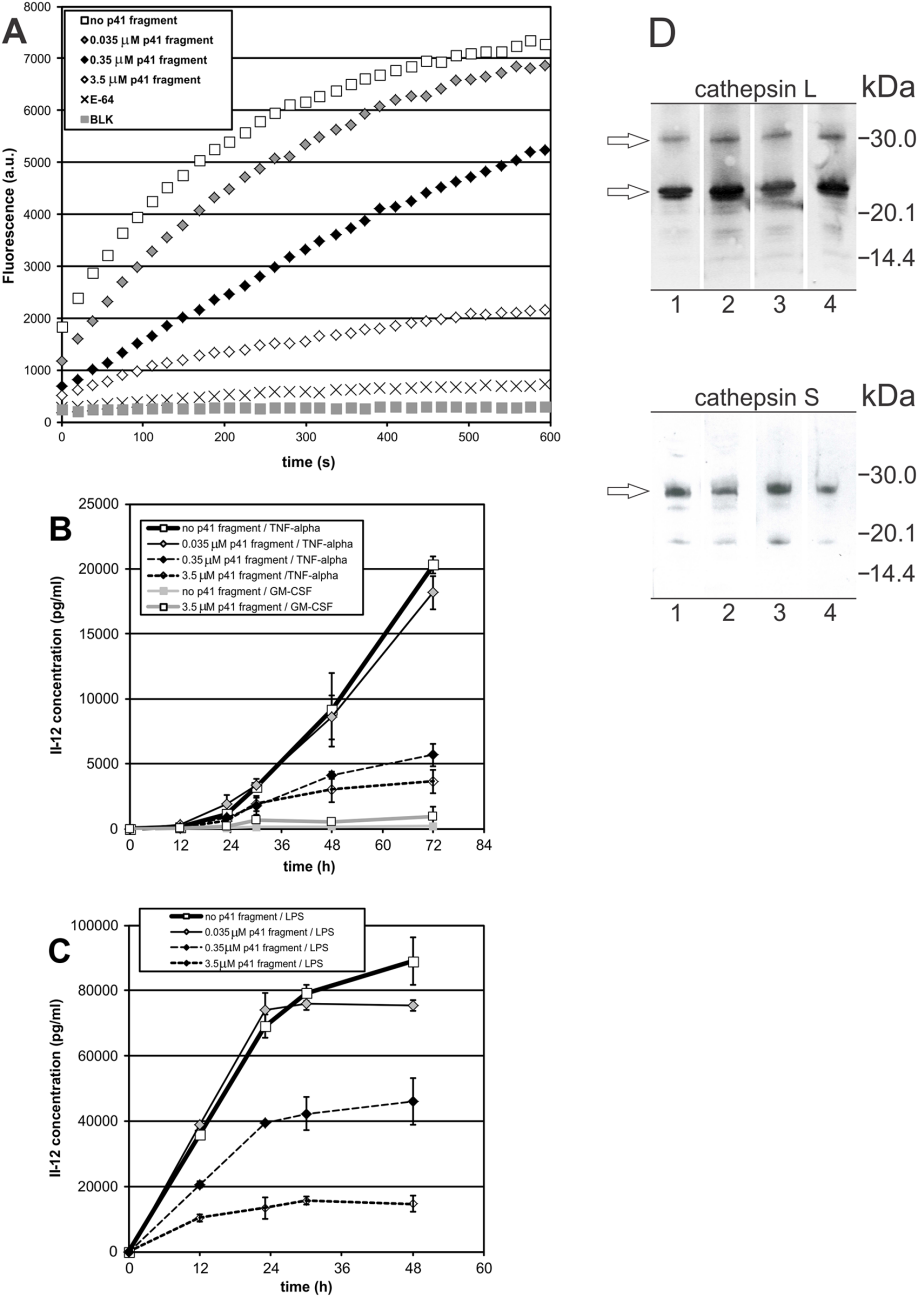
Effect of internalized p41 fragment on the proteolytic activity of cysteine proteases (A) and the secretion of IL-12/p70 (B, C). Samples (A): cell lysates of non-treated immature DC, immature DC after a 6-h incubation with p41 fragment (0.035 μM, 0.35 μM and 3.5 μM) and non-treated immature DC with 10 μM E-64. Fluorogenic substrate in buffer with DTT was used as blank (BLK). Representative measurements, each of three biological repetitions, are shown. Samples (B and C): cell free supernatants (culture media) of immature DC, preincubated with p41 fragment (0.035 μM, 0.35 μM and 3.5 μM) for 6 h prior to their maturation with TNF-α (B) or LPS (C). Non-treated cells (no preincubation with p41 fragment): immature DC, cultured in the presence of GM-CSF for three days (no maturation), DC matured with TNF-α, and DC matured with LPS. Pretreated but non-matured cells: immature DC, pretreated with 3.5 μM p41 fragment, and cultured in the presence of GM-CSF. IL-12 concentrations (in pg/ml) were measured in triplicate, average values ± SD are shown. (D) Immunolabelled cathepsins L and S in DC lysates. Samples (50 μg per well): (1) immature DC, (2) DC pretreated with 3.5 μM p41 fragment for 6 h, no LPS, (3) DC matured with LPS for 24 h, (4) DC pretreated with 3.5 μM p41 fragment for 6 h and matured with LPS for 24 h.

Cysteine protease activity in DC lysates (Fig 6) decreased after a 6-h incubation of cells with p41 fragment compared to that in non-treated immature DC grown in regular culture medium (Fig 6A). The greatest decline of fluorescence signal was observed when cells were preincubated with 3.5 μM p41 fragment. Nevertheless, cysteine protease activity was not abolished completely by the addition of p41 fragment. The diminished activity of cysteine proteases in treated DC was consistent with increased immunogold labelling of p41 epitope inside the endocytic pathway of treated DC. Addition of E-64 to a cell lysate led to diminished fluorescence signal, confirming that fluorogenic substrate was degraded by active cysteine proteases. Conversely, exogenous p41 fragment (3.5 μM), when preincubated with immature DC for 6 h prior to their maturation with LPS, did not significantly change the processing of immunolabelled cathepsin S or cathepsin L, when these two cysteine proteases were analyzed by Western blotting in lysates of treated DC (Fig 6D). Cathepsin L was immunolabelled at 24 kDa (double band) and at 30 kDa (single band). Cathepsin S was immunolabelled at 28 kDa. An unknown band below 20 kDa, reactive with anti-cathepsin S pAb, was detected.

### Secretion of IL-12 (p70) in non-treated DC

In non-treated TNF-α-matured DC, the secretion of IL-12 at the beginning of their maturation (0.1 ng/ml in the first 12 h) increased from 24 h onwards (Fig 6B). In contrast, the rate of secretion of IL-12 by LPS-stimulated DC cells was greater soon after their stimulation, i.e., 35.8±1.1 ng/ml was observed after 12 hours and 69.1±3.6 ng/ml after 24 h (Fig 6C). The IL-12 secretion following stimulation with LPS was four times greater than with TNF-α. In the presence of LPS, 89.0±7.4 ng/ml of IL-12 was secreted after 48 h, compared to TNF-α-matured DC which secreted 9.2±2.8 ng/ml after 48 h and 20.3±0.7 ng/ml by the end of a 3-day maturation. In the control experiment, immature DC, grown in the presence of GM-CSF, did not secrete significant amounts of IL-12 (Fig 6B). In the case of TNF-α matured DC, the secretion of IL-12 still continued to rise beyond 72 h whereas, in the case of LPS-matured DC, the secretion of IL-12 ceased in the second half of their maturation, from 24 h onwards (Fig 6C).

### Decreased secretion of IL-12 (p70) in p41 fragment-treated DC

Secretion of IL-12 during maturation of DC was compared with that by DC treated with p41 fragment prior to their maturation and by routinely matured DC described above. No significant decrease in IL-12 secretion followed the preincubation of immature DC with 0.035 μM p41 fragment. In contrast, in TNF-α-matured DC the secretion of IL-12 dropped considerably when 0.35 μM p41 Ii fragment was added, and amounted to only 5.7±0.9 ng/ml after 72 h. Addition of 0.35 μM p41 fragment prior to LPS maturation led to a secretion of IL-12 lower by one half (46.1±7.2 ng/ml after 48 h) than that in non-treated DC (89.0±7.4 ng/ml). Furthermore, more than 6-fold less IL-12 was secreted after 48 h when immature DC were preincubated with 3.5 μM p41 fragment and then stimulated with LPS. To summarize, addition of p41 fragment to the culture medium of immature DC (and its resultant internalization) led to decreased amounts of IL-12 secreted during their subsequent maturation with TNF-α or LPS.

In addition to the study with isolated p41 fragment, immature DC were pretreated with two exogenous recombinant Ii isoforms and the secretion of IL-12 followed on DC maturation with TNF-α (S4 Fig). Shorter Ii, that did not contain the inhibitory p41 fragment, was immunolabelled at 20 kDa. Longer Ii, with the inhibitory p41 fragment included in its luminal domain, was immunolabelled around 30 kDa (SDS-PAGE) and pI 5.3 (IEF). For the latter (3.5 μM p41 Ii), diminished secretion of IL-12 was observed; 4.6±0.7 ng/ml was secreted from treated cells, compared to non-treated cells which secreted 6.7±1.5 ng/ml after 48 hours (S4 Fig). The diminished secretion of IL-12 in cells, pretreated with p41 Ii, was much less profound when compared to the internalized exogenous p41 fragment (14 kDa) alone (Fig 6B). Shorter recombinant p31 Ii isoform did not notably change the secretion of IL-12 after 24 h, whereas, cells, pretreated with 3.5 μM p31 Ii, secreted 6.1±0.5 ng/ml when matured with TNF-α for 48 h. To summarize, addition of recombinant p41 Ii had a very limited effect on the secretion of IL-12 in DC stimulated with TNF-α, whereas the pretreatment of cells with p31 Ii resulted in a secretion of IL-12 comparable to that from non-treated cells when matured with TNF-α (S4 Fig).

Neither the addition of exogenous recombinant Ii with or without inhibitory p41 fragment (S4 Fig), nor the preincubation of cells with exogenous p41 fragment (Fig 6B) notably increased the otherwise low secretion of IL-12 from immature DC, cultured only in the presence of GM-CSF (without the stimulation with TNF-α or LPS).

### Nuclear translocation of NF-κB p65 was altered by p41 fragment

**Fig 7:**
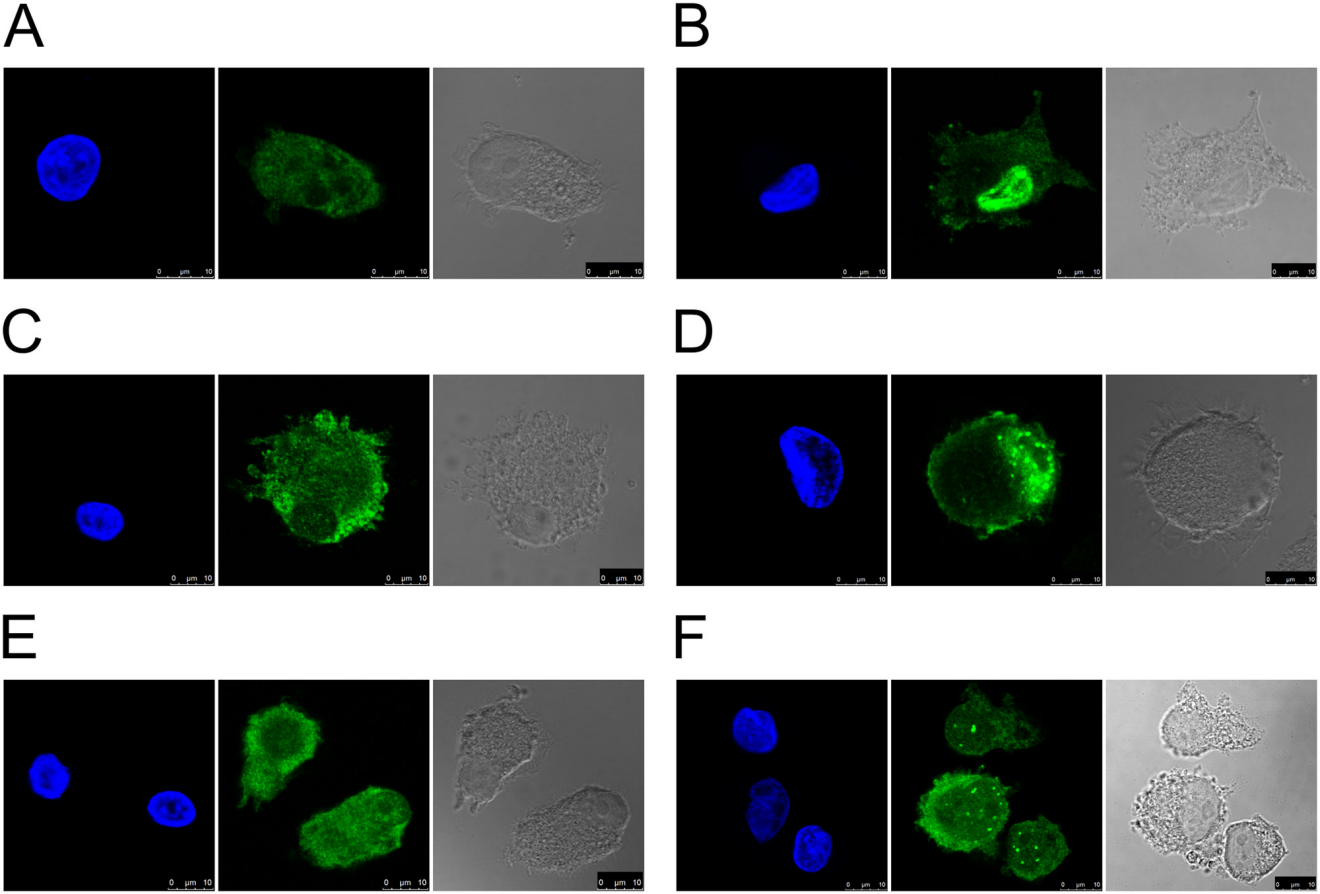
Translocation of NF-κB subunit p65 in immature DC treated with p41 fragment. Confocal images show localization of immunolabelled p65 in differentiated MUTZ-3 cells: (A) non-stimulated, (B) stimulated with LPS, (C) pretreated with 10 μM SN50 and stimulated with LPS, (D) pretreated with 10 μM SN50M and stimulated with LPS, (E) pretreated with 3.5 μM p41 fragment, (F) pretreated with pretreated with 3.5 μM p41 fragment and stimulated with LPS. Nuclei were stained with DAPI. Bars: 10 μm.

MUTZ-3 cell line was applied to determine the possible effect of internalized p41 fragment on NF-κB translocation when differentiated MUTZ-3 cells (immature DC) were stimulated with LPS. MUTZ-3 DCs, which have the great advantage of not being dependent on the donor material, express adequate immune-related transcripts, supporting their suitability in immune applications [57,58], and have been used in the translocation studies of NF-κB transcription factor subunits [51]. Here, IL-4-differentiated MUTZ-3 cells were treated with p41 fragment and the translocation of the p65 subunit analyzed (Fig 7). In non-stimulated cells p65 was localized mainly in the cytoplasm and was not increased in the nucleus, respectively (Fig 7A). After the stimulation of cells with LPS, translocation of p65 from the cytoplasm to the cell nucleus was observed and the cells exhibited strong nuclear staining in addition to the fluorescence signal in the cytoplasm (Fig 7B). The treatment of cells with 10 μM SN50, a cell-permeable inhibitor peptide of NF-κB nuclear translocation, reduced the fluorescence signal of immunolabelled p65 in the nucleus (Fig 7C), whereas SN50M (inactive control peptide) showed no effect on the nuclear translocation of p65, relative to LPS stimulation (Fig 7D). Yet, when differentiated MUTZ-3 cells were pretreated with exogenous p41 fragment, and then stimulated with LPS, the fluorescence signal of labelled p65 inside the nucleus was also reduced (Fig 7F), showing that the nuclear translocation of p65 on LPS stimulation has been affected. On the other hand, when cells were pretreated with p41 fragment, but not stimulated with LPS, the localization of immunolabelled p65 did not change and the fluorescence signal in the nucleus was not increased (Fig 7E).

## Discussion

Quantitation of the turnover rates of Ii showed that endogenous p41/p43 isoforms are more stable than the shorter p33/p35 isoforms, both in the endoplasmic reticulum and in Golgi [59]. Furthermore, it was reported that endogenous p41 Ii serves as a chaperone to help maintain a pool of mature cathepsin L in late-endocytic compartments of mouse bone marrow-derived macrophages [42]. We therefore speculated that endogenous p41 Ii, or its smaller fragments containing the characteristic inhibitory thyropin domain, would be extensively translocated into proteolytically active compartments, such as clustered perinuclear lysosomes in mature DC. Cathepsins S, L, and H, HLA-DM (Fig 2), and partially also MHC II (HLA-DR) (Fig 1), were labelled in these vesicles. However, as the results of confocal and immunogold electron microscopy show, this expectation has not been confirmed.

In contrast, the reduced immunogold labelling of endogenous p41 Ii in late endocytic compartments with multivesicular morphology (multivesicular bodies) (S1 Table) coincided with the absence of fluorescence signal of labelled endogenous p41 Ii in perinuclear LAMP 2^+^ lysosomes (Fig 2), both observed in mature DC, not pretreated with p41 fragment. Then again, it was reported that, in mature DC, phagocytosis induces the remodeling of clustered lysosomes, and regulated processing and presentation of particulate antigens by mature DC, via MHC II, still occurs [60]. It is possible that, in clustered perinuclear lysosomes of mature DC, the absence of endogenous p41 Ii and its shorter fragments containing the inhibitory thyropin domain would act in favor of those necessary proteolytic events that are performed by the active lysosomal cysteine proteases. Furthermore, mature DC are still capable of internalizing soluble antigens, processing them and presenting them to CD4^+^ T cells [61,62]. In contrast to the present cathepsins, it makes sense that their endogenous thyropin inhibitor would not be greatly retained inside the same clustered lysosomes, a lysosomal pool of HLA-DM and MHC II in mature DC, the latter already reported [62].

The question we asked was whether the situation described above would be similar to that when exogenous p41 fragment enters a cell from the extracellular milieu via the endocytic pathway. Would the exogenous thyropin inhibitor reach and then persist in acidic lysosomes and, consequently, change the cell proteolytic potential and, possibly, also other DC characteristics?

Two main issues have been addressed concerning the effects of added p41 fragment. p41 fragment, a representative of the thyropin family (I31, MEROPS database [63]), has been tested for its ability to inhibit the lysosomal cysteine proteases in DC prior to their maturation and, whether internalized p41 fragment alters the secretion of the cytokine IL-12 upon subsequent maturation of p41 fragment-treated immature DC.

Multivesicular bodies are the site of antigen processing and peptide binding to MHC II [26]. Significantly augmented distribution of characteristic p41 epitope within these late endocytic compartments with multivesicular morphology was confirmed and quantified by immunogold electron microscopy (S1 Table). p41 fragment *in vitro* inhibits most of the lysosomal cysteine cathepsins with K_i_ values in the picomolar or nanomolar range [38]. The reduced proteolytic activity of cysteine proteases in treated immature DC, reported here, further showed that targeted proteases (such as lysosomal cathepsins S, L and H) were accessed by internalized p41 fragment. The actual concentration of internalized p41 fragment in particular subcellular compartments could not be measured. However, the observed inhibition of cysteine protease activity in DC was dependent on the concentration of added p41 fragment and was consistent with the increased immunogold labelling frequency of p41 specific epitope inside treated immature DC. Concerning the protease capacity of DC, it makes a difference as to whether the inhibitory p41 fragment originates from the cell’s endogenous p41 Ii, or whether it reaches the cell acidic vesicles after it has been internalized from the extracellular milieu.

As reported in pancreatic β-cells [64], abnormal accumulation of pro-cathepsins B and L, instead of their active forms, following treatment with 20 μM irreversible synthetic inhibitors of cysteine proteases, suppresses the normal lysosomal degradation and processing of lysosomal enzymes, all this leading to lysosomal dysfunction. As for DC, like APC, this would also mean loss of their ability to successfully process incoming antigens. We could not confirm that internalized p41 fragment, a reversible inhibitor, when applied to immature DC in a 5.7-times lower micromolar concentration, would also vastly change the processing of cathepsins L and S, the two selected lysosomal proteases.

Activated DC produce a variety of cytokines whose type and levels of expression direct T-cell differentiation towards a Th1 or a Th2 immune response. The IL-12 family possesses a significant role and IL-12 (p70), a heterodimeric cytokine consisting of p35 and p40 subunits, is involved in the differentiation of naive CD4^+^ T cells to Th1 cells [65]. In our study the concentrations of IL-12 (active p70 form) secreted on maturation of non-treated DC were compared with those secreted by DC treated with p41 fragment. In comparison to LPS maturation, and in accordance with reported results [50, 66], lower amounts of IL-12 were observed when non-treated DC were matured with TNF-α.

Interestingly, after p41 fragment is internalized into immature DC, both types of their subsequent maturation, i.e., with LPS or with TNF-α, resulted in greatly diminished secretion of IL-12. How the p41 fragment, recognized as an inhibitor of lysosomal cysteine proteases [38,39], affects IL-12 secretion in treated human DC is not clear. No difference in IL-12 secretion, in response to LPS, was observed between bone marrow-derived DC from cathepsin L-deficient mice and from wild type mice [67]. On the other hand, selected parasite cystatin-type inhibitors, such as sialostatin L from the tick *I*. *scapularis*, interfere with the TLR-mediated release of IL-12 in DC [68].

We report here that exogenous p41 fragment affects the translocation of NF-κB subunit p65 from the cytosol to the nucleus in LPS-stimulated DC. The level of NF-κB transcription factor (p65/p50 heterodimer) in the nucleus is closely related to the expression of proinflammatory genes, among which also regulates the expression of IL-12 in DC [69–71]. Further, numerous examples of bacterial interference and immune evasion mechanisms at various steps in the TLR-NF-κB pathway have been reported (reviewed in [72]). Here we propose that the diminished secretion of IL-12 in LPS-stimulated and p41 fragment-pretreated DC is linked to this exogenous inhibitor interference with the TLR-mediated NF-κB signaling pathway. Conversely, TLR signaling cascades don’t regulate the cysteine cathepsins directly, rather their activities were reported to be regulated indirectly, i.e., by cytokines secreted in response to TLR activation [73]. Cytokines induced by the MyD88-dependent and MyD88-independent signaling cascades, associated with LPS/TLR4 stimulation, increased the proteolytic activities of cysteine cathepsins L and S in macrophages without changing their mRNA expression [73]. It would be interesting to further explore how a thyropin type inhibitor such as p41 fragment may influence a MyD88-dependent pathway, activated on the stimulation of TLR4. As reported for mouse DC [74], the release of IL-12 by DC activated by TLR ligation is dependent on MyD88 signaling.

So far it is only possible to speculate as to whether p41 fragment exhibits intrinsic structural features that would enable this thyropin to be engaged in receptor binding. In our DC model fluorescently labelled p41 fragment did not bind exclusively to the plasma membrane of treated immature DC (Fig 4D). However, the diminished production of pro-inflammatory cytokine IL-12 by DC would skew CD4^+^ T cells away from a Th1 immune response [75,76]. Conversely, the supplement of mycobacteria-infected macrophages with IL-12, together with neutralization of IL-27, induces the formation of the mature (and active) lysosomal aspartic protease cathepsin D [77]. Further, cathepsin B, another lysosomal cysteine protease, has been proposed to play a role in IL-12 expression, down-regulating the Th1 response during *Leishmania major* infection of mice [67].

The inhibition of proteolytic activity of lysosomal cysteine proteases in our model immature DC coupled with the diminished capability of DC to produce IL-12 on succeeding maturation, supports the immunomodulatory potential of the thyropin from p41 Ii. Conceivably, this would apply also to other putative thyropin-type inhibitors when acting on host DC. For example, in tick saliva several new putative proteins with the thyroglobulin type-1 domain, a main characteristic of thyropin-type inhibitors, have been reported on proteomic [45,46], sialotranscriptomic [78,79] and genomic analysis [80]. Moreover, the CBU0898 locus of *Coxiella burnetti* possesses a sequence homologous to the thyroglobulin type-1 domain [81,82]. Interestingly, this bacterium, which is also transmitted by tick saliva, successfully propagates inside acidic vesicles in host APC, i.e., DC [83] and macrophages [84]. Clearly, the proposed thyropin inhibitory capability of these salivary and bacterial proteins needs to be proven and its putative role towards host APC precisely evaluated. Hypothetically, after thyropin internalization, the proteolytic potential of a host cell may be decreased and a pathogen (or a parasite) provided with better conditions to survive and propagate. We speculate that an additional amount of the thyropin inhibitor, in addition to endogenous p41 Ii, might completely change the proteolysis and effective processing of antigens. Among others, lysosomal cathepsin S would also be blocked [38] and thus non-active for a crucial step in Ii processing, i.e., for αβ-CLIP complex formation [85].

The introduction of additional inhibitory p41 fragment has been shown to change the cell proteolytic potential as well as the secretion of regulatory molecules such as IL-12. Added p41 fragment entered the endocytic pathway of immature DC and contributed markedly to the observed immunogold labelling frequency of p41 Ii-specific epitope, in particular in late endocytic compartments (multivesicular bodies) where antigen processing and binding to MHC II takes place. Internalized p41 fragment acted effectively on cysteine proteases inside human immature DC, but whether the reduction of IL-12 secretion was directly linked to the diminished proteolytic activity of one or more cysteine proteases remains to be elucidated.

## Supporting Information

S1 Fig Characterization of DC. The phenotype of immature DC (column A) and of mature DC after 3 days of maturation with TNF-α (column B). Continuous-line histograms: CD14, HLA-DR, CD1a, CD40, CD54, CD80, CD83 and CD86. Shadowed histograms: negative controls (binding of irrelevant isotype-matched antibody). Confocal images (D–G, I, J): the uptake of fluorescent Alexa Fluor 546-labelled dextran. TEM image (K): ultrastructure of immature DC. Bars: 15 μm (C–J), 5 μm (K).
(PDF)

S2 Fig Specificity of anti-p41 Ii mAb against the epitope within inhibitory p41 fragment. SDS-PAGE (A, B, E) and native PAGE (C, D) were performed and proteins labelled with anti-p41 Ii mAb (A, C, E) or stained with silver (B, D). Samples A, B: (1) p41 fragment/cathepsin L complex, (2) procathepsin L, (3) p41 Ii, (4) p41 fragment. All samples but one (1) were reduced with DTT and boiled prior to SDS-PAGE. Samples C, D: (1) p41 fragment, (2) p41 fragment, preincubated with N-Glycosidase F, (3) p41 fragment/cathepsin L complex, (4) p41 fragment/cathepsin L complex, preincubated with N-Glycosidase F. Samples E: (1) p41 fragment, (2) lymph node lysate. (F) p41 Ii-positive cells in lymph node paracortex. Bar: 30 μm. The position of p41 fragment in human Ii isoforms is indicated. C–cytoplasmic, M–transmembrane, L–luminal. ST–standards.
(PDF)

S3 Fig Separation of fluorescently labelled p41 fragment from the unreacted Alexa Fluor 488 dye. Fluorescence was measured after gel filtration (A) and after dialysis and membrane filtration (C, D, E, F). Fractions containing conjugated p41 fragment (A, C, E) were compared to those containing unreacted dye (D, F) and to PBS buffer before dialysis (B). Confocal and DIC image: DC, preincubated with filtrate F (residual unreacted dye), bars: 15 μm.
(PDF)

S4 Fig Characterization of two recombinant Ii isoforms (A, B) and their effect on the secretion of IL-12/p70 (C, D). SDS-PAGE (A) and IEF (B) separated proteins stained with Coomassie dye (standards, A1), silver (A2, A3, B1) or blotted to membrane and labelled with anti-Ii (LN2) mAb (A4, A5). Samples: recombinant Ii with inhibitory p41 fragment (A1, A2, A4, B1), recombinant Ii without inhibitory p41 fragment (A3, A5). ST–standards. Arrows indicate two Ii isoforms as monomers. Minor portions of both recombinant Ii were labelled above 30 kDa and 43 kDa (bands represent dimers). (C, D) IL-12 in cell free supernatants (culture media) of immature DC, preincubated with recombinant p41 Ii (C) or p31 Ii (D) for 6 h prior to their maturation with TNF-α. Non-treated cells are: immature DC, cultured in the presence of GM-CSF (no maturation), and DC, matured with TNF-α. Pretreated non-matured cells are: immature DC, pretreated with Ii, and cultured in the presence of GM-CSF. IL-12 concentrations (in pg/ml) were measured in triplicate, average values ± SD are shown.
(PDF)

S5 Fig Specificity of anti-cathepsin L and anti-cathepsin S polyclonal antibodies. Immunolabelled recombinant human cathepsin L–heavy chain (A) and cathepsin S (B), both expressed in *Pichia pastoris*. ST–standards.
(PDF)

S1 Table Distributions of immunogold labelled p41 Ii and cathepsin S and chi-squared (X^2^) test. Values represent observed and expected (in brackets) numbers of 10-nm gold particles in immature DC, mature DC and in immature DC treated with 3.5 μM inhibitory p41 fragment for 6 h.
(PDF)
