# Supplementary figures and images for: Exogenous Thyropin from p41 Invariant Chain Diminishes Cysteine Protease Activity and Affects IL-12 Secretion during Maturation of Human Dendritic Cells
Source: PLoS One. 2016 Mar 9;11(3):e0150815. doi: 10.1371/journal.pone.0150815 (PMC4784741; doi:10.1371/journal.pone.0150815)

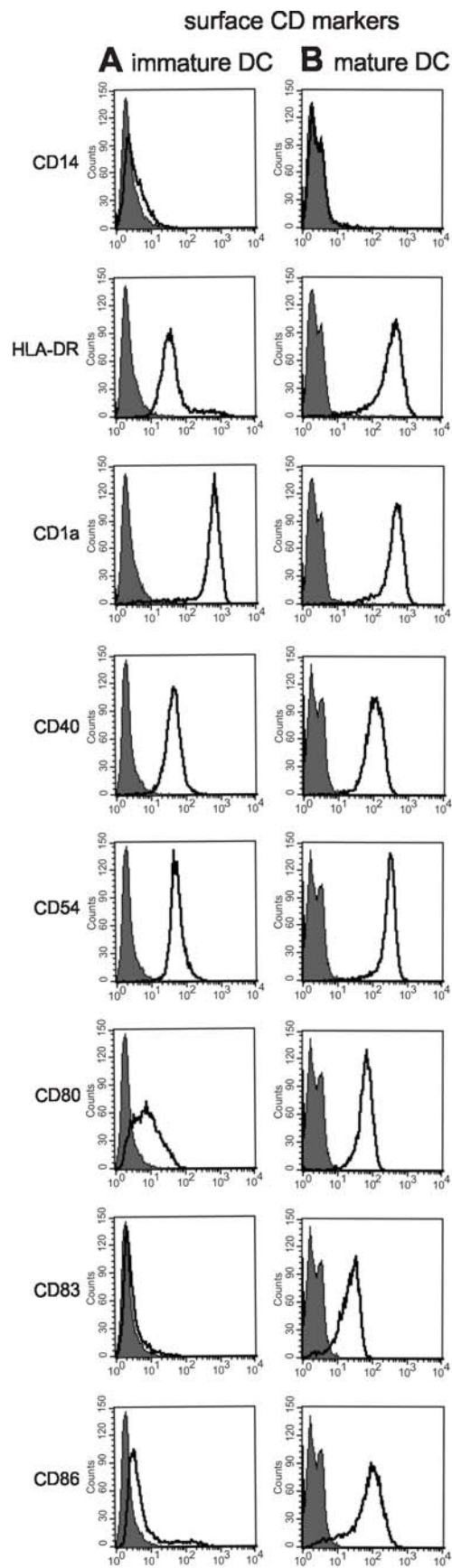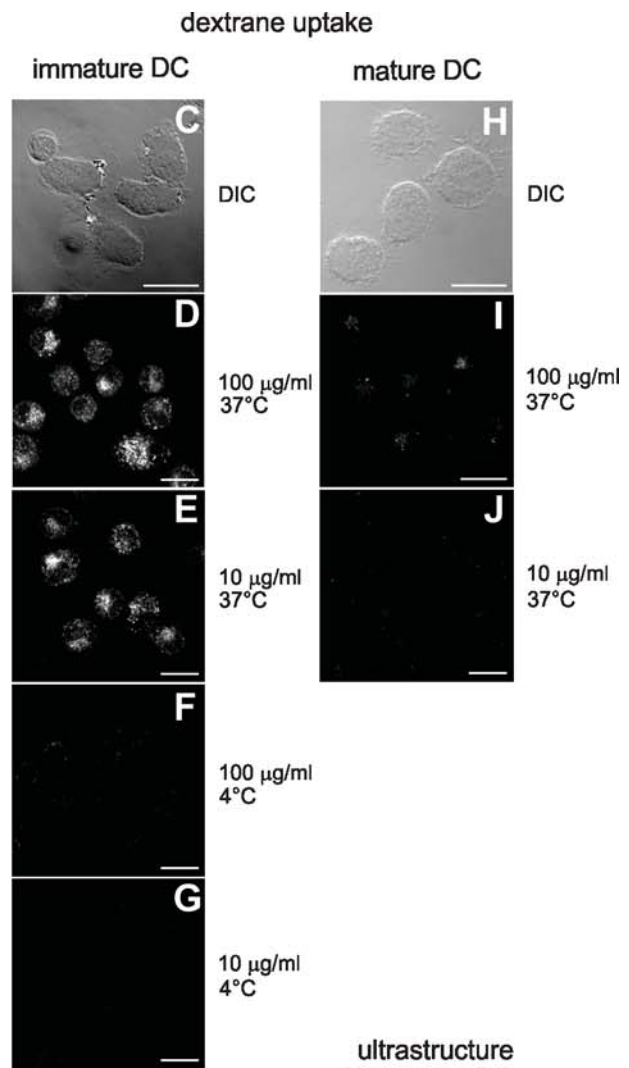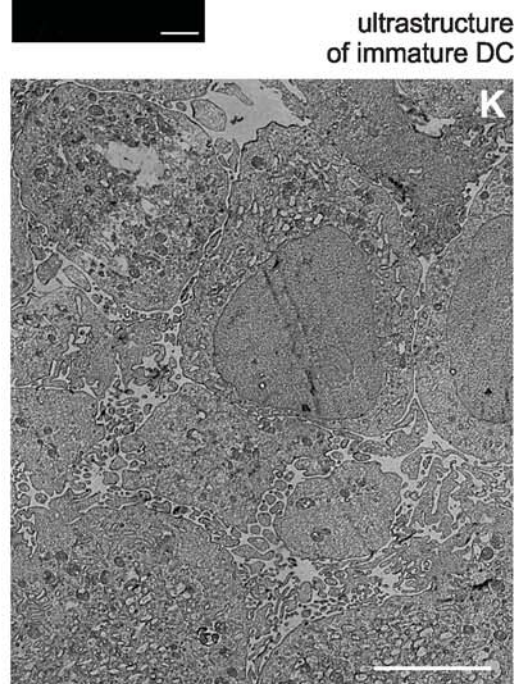

Supplement: S1 Fig — The phenotype of immature DC (column A) and of mature DC after 3 days of maturation with TNF-α (column B). Continuous-line histograms: CD14, HLA-DR, CD1a, CD40, CD54, CD80, CD83 and CD86. Shadowed histograms: negative controls (binding of irrelevant isotype-matched antibody). Confocal images (D–G, I, J): the uptake of fluorescent Alexa Fluor 546-labelled dextran. TEM image (K): ultrastructure of immature DC. Bars: 15 μm (C–J), 5 μm (K). (PDF) [file pone.0150815.s001.pdf]

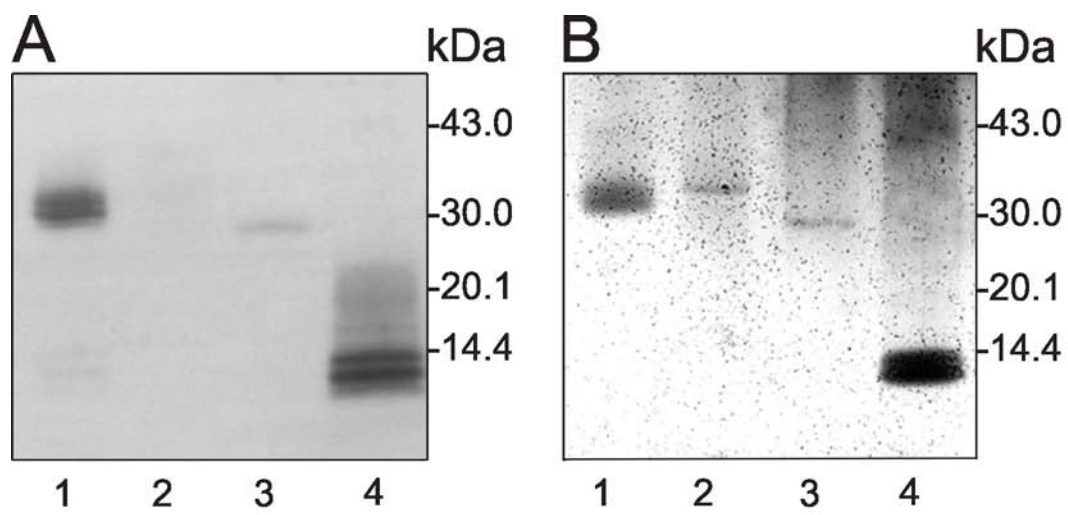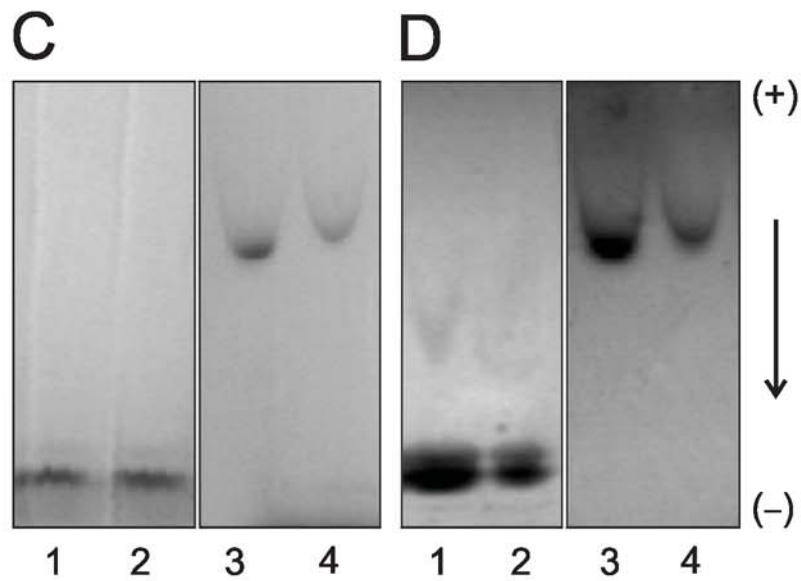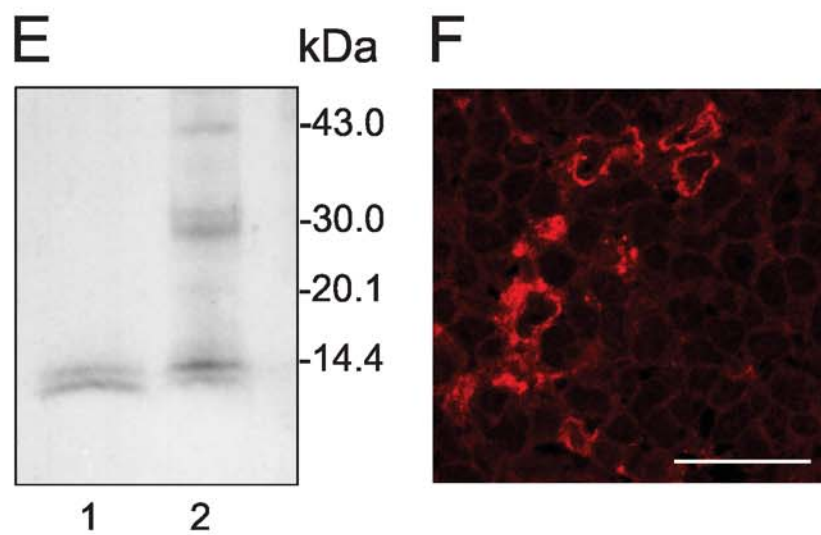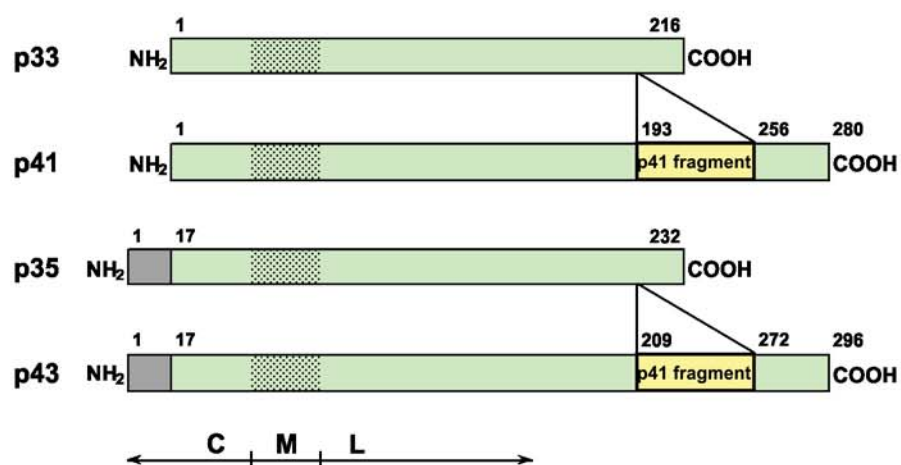

Supplement: S2 Fig — SDS-PAGE (A, B, E) and native PAGE (C, D) were performed and proteins labelled with anti-p41 Ii mAb (A, C, E) or stained with silver (B, D). Samples A, B: (1) p41 fragment/cathepsin L complex, (2) procathepsin L, (3) p41 Ii, (4) p41 fragment. All samples but one (1) were reduced with DTT and boiled prior to SDS-PAGE. Samples C, D: (1) p41 fragment, (2) p41 fragment, preincubated with N-Glycosidase F, (3) p41 fragment/cathepsin L complex, (4) p41 fragment/cathepsin L complex, preincubated with N-Glycosidase F. Samples E: (1) p41 fragment, (2) lymph node lysate. (F) p41 Ii-positive cells in lymph node paracortex. Bar: 30 μm. The position of p41 fragment in human Ii isoforms is indicated. C–cytoplasmic, M–transmembrane, L–luminal. ST–standards. (PDF) [file pone.0150815.s002.pdf]

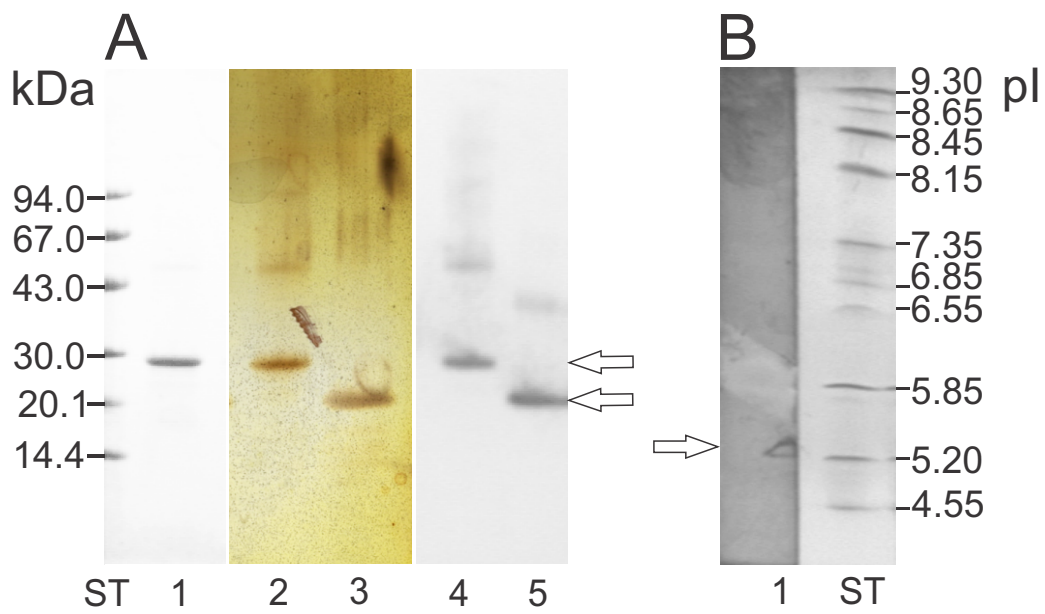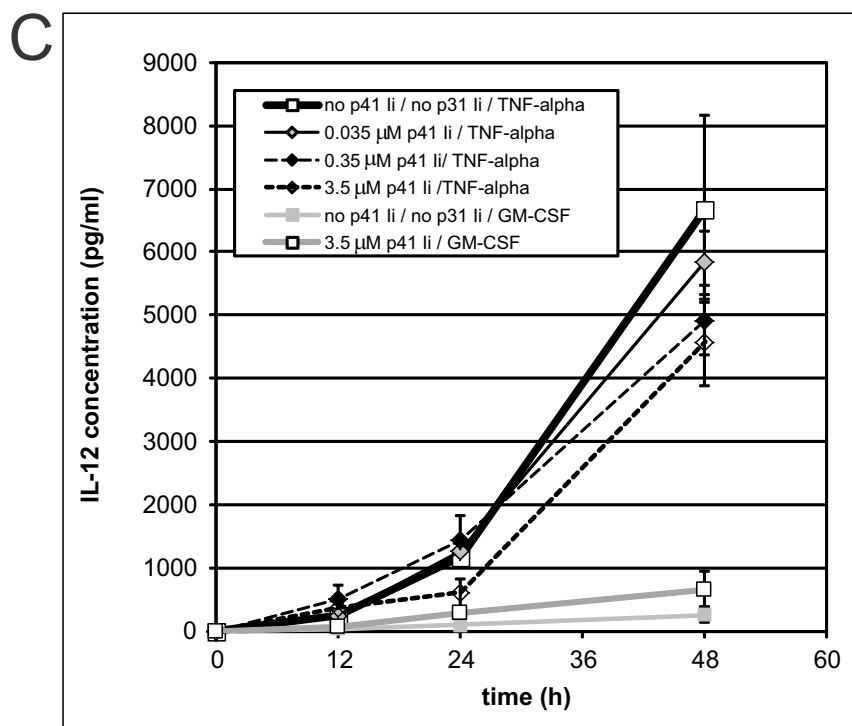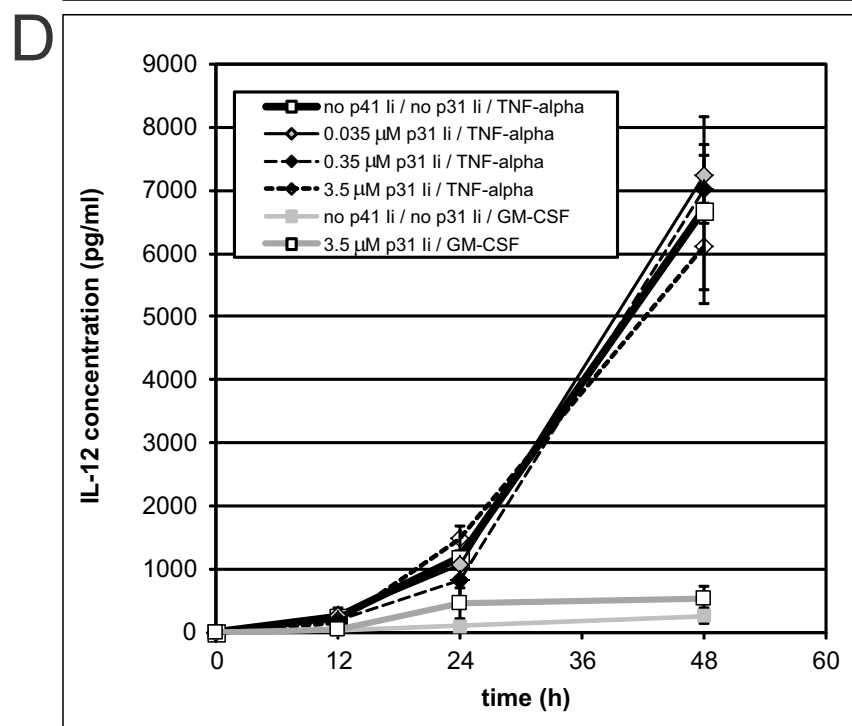

Supplement: S4 Fig — SDS-PAGE (A) and IEF (B) separated proteins stained with Coomassie dye (standards, A1), silver (A2, A3, B1) or blotted to membrane and labelled with anti-Ii (LN2) mAb (A4, A5). Samples: recombinant Ii with inhibitory p41 fragment (A1, A2, A4, B1), recombinant Ii without inhibitory p41 fragment (A3, A5). ST–standards. Arrows indicate two Ii isoforms as monomers. Minor portions of both recombinant Ii were labelled above 30 kDa and 43 kDa (bands represent dimers). (C, D) IL-12 in cell free supernatants (culture media) of immature DC, preincubated with recombinant p41 Ii (C) or p31 Ii (D) for 6 h prior to their maturation with TNF-α. Non-treated cells are: immature DC, cultured in the presence of GM-CSF (no maturation), and DC, matured with TNF-α. Pretreated non-matured cells are: immature DC, pretreated with Ii, and cultured in the presence of GM-CSF. IL-12 concentrations (in pg/ml) were measured in triplicate, average values ± SD are shown. (PDF) [file pone.0150815.s004.pdf]

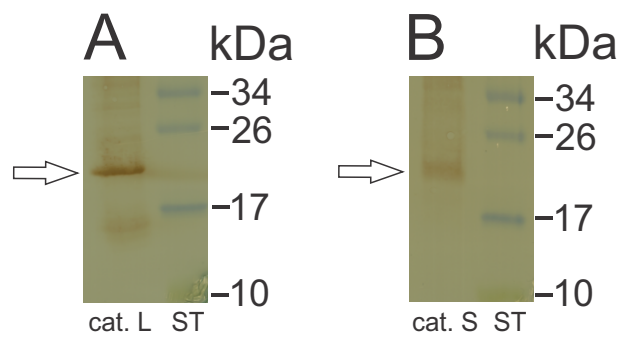

Supplement: S5 Fig — Immunolabelled recombinant human cathepsin L–heavy chain (A) and cathepsin S (B), both expressed in Pichia pastoris. ST–standards. (PDF) [file pone.0150815.s005.pdf]
