# Supplementary material for: Exogenous Thyropin from p41 Invariant Chain Diminishes Cysteine Protease Activity and Affects IL-12 Secretion during Maturation of Human Dendritic Cells
Source: PLoS One. 2016 Mar 9;11(3):e0150815. doi: 10.1371/journal.pone.0150815 (PMC4784741; doi:10.1371/journal.pone.0150815)
Supplement: S3 Fig — Fluorescence was measured after gel filtration (A) and after dialysis and membrane filtration (C, D, E, F). Fractions containing conjugated p41 fragment (A, C, E) were compared to those containing unreacted dye (D, F) and to PBS buffer before dialysis (B). Confocal and DIC image: DC, preincubated with filtrate F (residual unreacted dye), bars: 15 μm. (PDF) [file pone.0150815.s003.pdf]

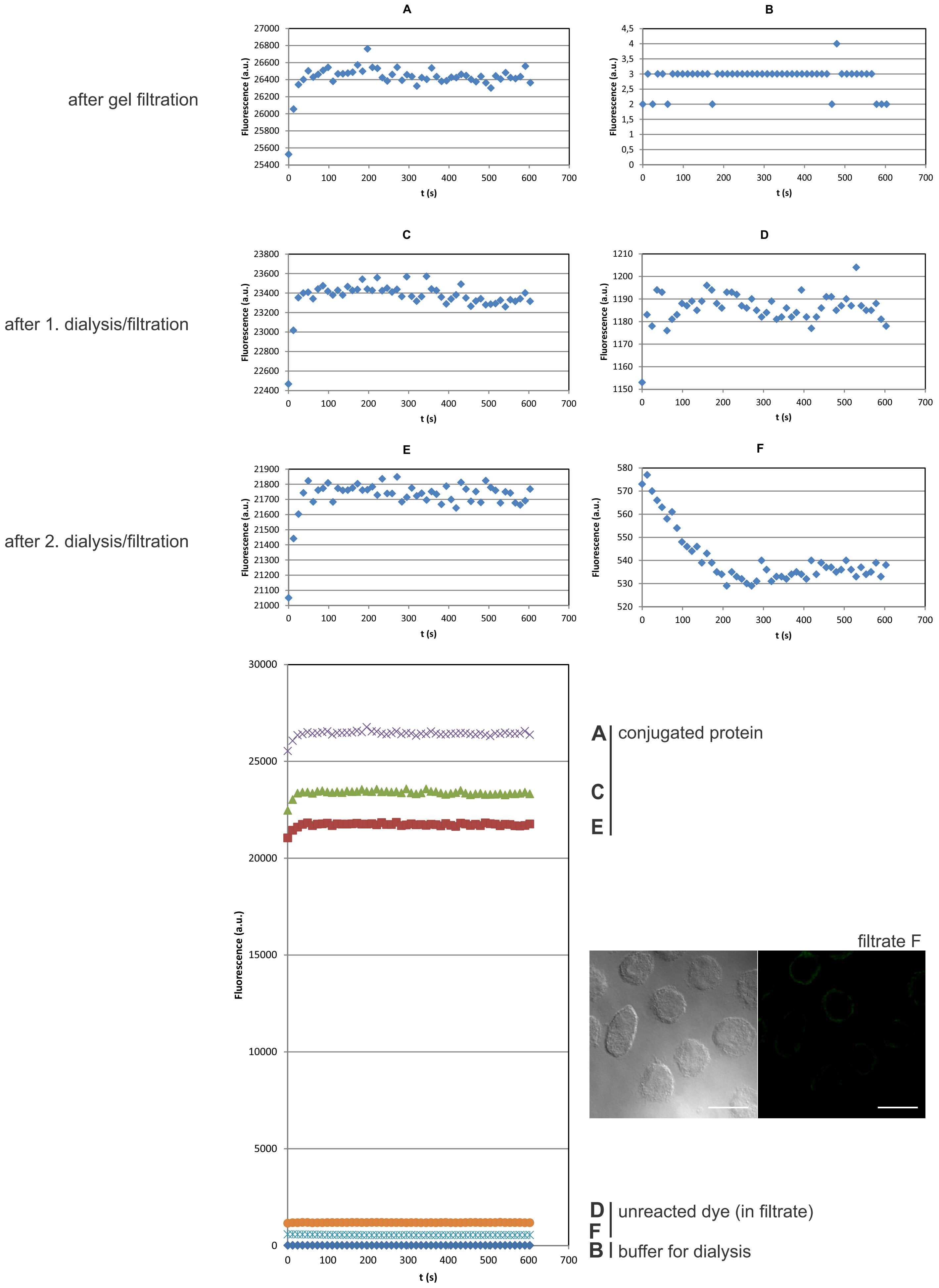
after gel filtration
after 1. dialysis/filtration
after 2. dialysis/filtration
A
B
C
D
E
F
Fluorescence (a.u.)
t (s)
A conjugated protein
C
E
filtrate F
D unreacted dye (in filtrate)
F
B buffer for dialysis
