# Supplementary material for: Exogenous Thyropin from p41 Invariant Chain Diminishes Cysteine Protease Activity and Affects IL-12 Secretion during Maturation of Human Dendritic Cells
Source: PLoS One. 2016 Mar 9;11(3):e0150815. doi: 10.1371/journal.pone.0150815 (PMC4784741; doi:10.1371/journal.pone.0150815)
Supplement: S1 Table — Values represent observed and expected (in brackets) numbers of 10-nm gold particles in immature DC, mature DC and in immature DC treated with 3.5 μM inhibitory p41 fragment for 6 h. (PDF) [file pone.0150815.s006.pdf]

A

subcellular compartments	counted gold particles associated with: p41 Ii, immature DC		p41 Ii, mature DC		rows total	chi-squared values: immature DC	chi-squared values: mature DC
small vesicles	82	(91.0)	100	(91.0)	**182**	0.89	0.89
Golgi	20	(17.0)	14	(17.0)	**34**	0.53	0.53
endoplasmic reticulum and nuclear membrane	18	(18.0)	18	(18.0)	36	0.00	0.00
multivesicular bodies	40	(36.0)	32	(36.0)	**72**	0.44	0.44
small vesicles near plasma membrane	6	(7.0)	8	(7.0)	**14**	0.14	0.14
plasma membrane	10	(9.0)	8	(9.0)	**18**	0.11	0.11
other incl. nucleus, cytoplasm and mitochondria	24	(22.0)	20	(22.0)	44	0.18	0.18
**Columns total**	**200**		**200**		**400**	**4.58***	

*For a total chi-squared value (X^2) of 4.58 and 6 degrees of freedom (P=0.596) these distributions are not significantly different.
Degrees of freedom: (7 − 1 rows) by (2 − 1 columns) = 6.

B

subcellular compartments	counted gold particles associated with: p41 Ii, immature DC		p41 Ii, mature DC		added p41 fragment		rows total	chi-squared values: immature DC	chi-squared values: mature DC	chi-squared values: added p41 fragment
small vesicles	82	(83.7)	100	(83.7)	69	(83.7)	**251**	0.04	3.17	2.58
Golgi	20	(13.0)	14	(13.0)	5	(13.0)	**39**	3.77	0.08	4.92
endoplasmic reticulum and nuclear membrane	18	(13.7)	18	(13.7)	5	(13.7)	**41**	1.35	1.35	5.53
multivesicular bodies	40	(46.7)	32	(46.7)	68	(46.7)	**140**	0.96	4.63	9.71
small vesicles near plasma membrane	6	(12.0)	8	(12.0)	22	(12.0)	**36**	3.00	1.33	8.33
plasma membrane	10	(11.7)	8	(11.7)	17	(11.7)	**35**	0.25	1.17	2.40
other incl. nucleus, cytoplasm and mitochondria	24	(19.3)	20	(19.3)	14	(19.3)	58	1.15	0.03	1.46
**Columns total**	**200**		**200**		**200**		**600**		**57.21****	

**For a total chi-squared value (X^2) of 57.21 and 12 degrees of freedom (P<0.001) these distributions are significantly different.
Degrees of freedom: (7 − 1 rows) by (3 − 1 columns) = 12.

C

subcellular compartments	counted gold particles associated with: cathepsin S, immature DC		cathepsin S, mature DC		rows total	chi-squared values: immature DC	chi-squared values: mature DC
small vesicles	37	(29.4)	22	(29.7)	**59**	1.97	2.00
Golgi, endoplasmic reticulum and nuclear membrane	7	(5.0)	3	(5.0)	**10**	0.80	0.80
multivesicular bodies	142	(149.8)	159	(151.3)	**301**	0.41	0.39
small vesicles near plasma membrane and plasma membrane	7	(7.5)	8	(7.5)	**15**	0.03	0.03
other incl. nucleus, cytoplasm and mitochondria	8	(9.5)	11	(9.6)	19	0.24	0.20
**Columns total**	**201**		**203**		**404**	**6.87*****	

***For a total chi-squared value (X^2) of 6.87 and 4 degrees of freedom (P=0.141) these distributions are not significantly different.
Degrees of freedom: (5 − 1 rows) by (2 − 1 columns) = 4.
